# The non-mevalonate pathway requires a delicate balance of intermediates to maximize terpene production

**DOI:** 10.1007/s00253-024-13077-7

**Published:** 2024-02-29

**Authors:** Indu Raghavan, Rosheena Juman, Zhen Q. Wang

**Affiliations:** Department of Biological Sciences, University at Buffalo, the State University of New York, 653 Cooke Hall, Buffalo, New York, NY14260 USA

**Keywords:** Methylerythritol phosphate pathway, 1-deoxy-D-xylulose 5-phosphate, Feedback inhibition, Flux balance, Geraniol

## Abstract

**Abstract:**

Terpenes are valuable industrial chemicals whose demands are increasingly being met by bioengineering microbes such as *E. coli*. Although the bioengineering efforts commonly involve installing the mevalonate (MVA) pathway in *E. coli* for terpene production, the less studied methylerythritol phosphate (MEP) pathway is a more attractive target due to its higher energy efficiency and theoretical yield, despite its tight regulation. In this study, we integrated an additional copy of the entire MEP pathway into the *E. coli* genome for stable, marker-free terpene production. The genomically integrated strain produced more monoterpene geraniol than a plasmid-based system. The pathway genes’ transcription was modulated using different promoters to produce geraniol as the reporter of the pathway flux. Pathway genes, including *dxs*, *idi*, and *ispDF*, expressed from a medium-strength promoter, led to the highest geraniol production. Quantifying the MEP pathway intermediates revealed that the highest geraniol producers had high levels of isopentenyl pyrophosphate (IPP) and dimethylallyl pyrophosphate (DMAPP), but moderate levels of the pathway intermediates upstream of these two building blocks. A principal component analysis demonstrated that 1-deoxy-D-xylulose 5-phosphate (DXP), the product of the first enzyme of the pathway, was critical for determining the geraniol titer, whereas MEP, the product of DXP reductoisomerase (Dxr or IspC), was the least essential. This work shows that an intricate balance of the MEP pathway intermediates determines the terpene yield in engineered *E. coli*. The genetically stable and intermediate-balanced strains created in this study will serve as a chassis for producing various terpenes.

**Key points:**

*• Genome-integrated MEP pathway afforded higher strain stability*

*• Genome-integrated MEP pathway produced more terpene than the plasmid-based system*

*• High monoterpene production requires a fine balance of MEP pathway intermediates*

**Supplementary Information:**

The online version contains supplementary material available at 10.1007/s00253-024-13077-7.

## Introduction

Terpenes are the largest class of plant secondary metabolites with enormous commercial and medicinal values (Tetali 2019). They are often extracted from plants at low yields. Manufacturing valuable terpenes from fast-growing microbes such as *Escherichia coli* and *Saccharomyces cerevisiae* holds excellent promise for the bioeconomy (Wang et al. 2018). Two biosynthetic routes of terpenes coexist in nature: the mevalonate (MVA) pathway in eukaryotes and archaea and the methyl erythritol phosphate (MEP) pathway, also called the non-mevalonate pathway, in bacteria and plant chloroplasts (Kuzuyama and Seto 2012). The well-studied MVA pathway is a frequent target for microbial expression to produce terpenes (Alonso-Gutierrez et al. 2013; Anthony et al. 2009; Harada et al. 2009; Kim et al. 2016; Martin et al. 2003; Rodríguez-Villalón et al. 2008; Wang et al. 2010; Wang et al. 2016; Westfall et al. 2012; Yoon et al. 2009). However, the MEP pathway is more attractive because of the higher maximum stoichiometric yield, better redox balance, and lower energy consumption (Gruchattka et al. 2013). Nevertheless, the heterologous expression of the MEP pathway in baker’s yeast is challenging due to the difficulties associated with expressing [Fe-S]-containing enzymes, in particular 4-hydroxy-3-methyl-but-2-enyl pyrophosphate synthase or IspG, in this eukaryotic host (Carlsen et al. 2013; Kirby et al. 2016; Partow et al. 2012). Thus, engineering the MEP pathway in a bacterial host such as *E. coli* is advantageous because of its ability to express functional [Fe-S]-containing enzymes.

Plasmids are frequently used to express metabolic pathway enzymes in *E. coli* due to their ease of handling. However, inserting overexpressed genes into the genome avoids metabolic burden, allows antibiotic-free cultivation, confers greater genetic stability, and maintains a precise copy number (Striedner et al. 2010; Tyo et al. 2009). Moreover, overexpressing the MEP pathway by genome integration will liberate bioengineers from repetitive engineering of the MEP pathway to increase terpene flux: researchers can focus instead on downstream engineering by transforming plasmids encoding various prenyltransferases and terpene synthases into the genome-integrated strain to produce target terpenes. However, existing literature only reports the genomic integration of the partial MEP pathway or modulation of the promoter and RBS strengths of native MEP genes (Ajikumar et al. 2010; Li et al. 2015; Su et al. 2020; Wang et al. 2009; Yuan et al. 2006; Zhao et al. 2013a). The recent advent of the CRISPR/Cas9 mediated genome insertion in *E. coli* enables stable integration of the entire MEP pathway and the combinatorial screening of promoters to maximize terpene production.

In this study, we overexpressed the *E. coli* MEP genes by inserting an additional copy of the entire eight-gene pathway into its genome. The performance of the strains was assessed by producing a monoterpene, geraniol. The genome-integrated strain exhibited excellent genetic stability, higher cell viability, and higher geraniol titer than a strain expressing the entire pathway on plasmids. We modulated the expression of the genome-integrated pathway by creating a combinatorial library of 27 strains with promoters of different strengths. The observation that a medium-strength promoter led to the maximum geraniol titer prompted us to analyze the MEP pathway intermediates. Quantifying intracellular MEP pathway intermediates in the library revealed that not only the levels of IPP and DMAPP but also a delicate balance of upstream intermediates was vital to terpene yields.

## Materials and methods

### Strains, media, and culturing conditions

All the strains used in this study are listed in Table S1. The strain DH5α was used for cloning all the constructs except for the final assemblies of the MEP gene cassettes, for which MG1655(DE3) was used. Chemically competent cells were transformed with plasmids using heat shock. Cells were plated on Luria-Bertani (LB) agar (10 g/L peptone, 5g/L yeast extract, 5 g/L NaCl, 12 g/L agar) (Thermo Fisher Scientific, Waltham, MA, USA) containing the respective antibiotics and incubated at 37 °C overnight.

Single colonies were inoculated in 3 mL LB broth (10 g/L peptone, 5 g/L yeast extract, and 5 g/L NaCl) (Thermo Fisher Scientific, Waltham, MA, USA) containing the respective antibiotics at 37 °C at 220 rpm overnight. The overnight seed cultures were used to inoculate shake flasks containing 25 mL Terrific Broth (TB) (12 g/L tryptone, 24 g/L yeast extract, 2.2 g/L KH_2_PO_4_, and 9.4 g/L K_2_HPO_4_) (Thermo Fisher Scientific, Waltham, MA, USA) with 1.5% (w/v) glucose (MP Biomedicals, Santa Ana, CA, USA) and the respective antibiotics, at an initial OD_600_ of 0.05. The cultures were grown at 37 °C at 220 rpm and induced at an OD_600_ of 1–1.2 using 1 mM isopropylthio-β-galactoside (IPTG) (GoldBio, St. Lois, MO, USA). Cultures for fluorescence imaging were grown aerobically at 16° for 16 h post induction. The cultures for geraniol and metabolite quantification were grown at 30 °C post induction in microaerobic conditions by sealing the flasks with a parafilm. An additional 1% (w/v) glucose was added to the cultures at 24 h after induction, after which flasks were re-parafilmed and grown at 30 °C and harvested at various time points, as indicated in the figures.

### Molecular cloning

All plasmids used in this study are listed in Table S2, which also includes the GenBank and EcoCyc accession numbers of all cloned genes. Primers are listed in Tables S3 and S4. The ribosome binding sites for all the genes cloned in this study, except *lacI* and the antibiotic resistance genes, were designed using the RBS calculator (Salis 2011) and included in the primers. The promoters, RBSs, and terminators for *lacI* and the antibiotic resistance genes were amplified with the respective coding regions from source plasmids.

**Fig. 1:**
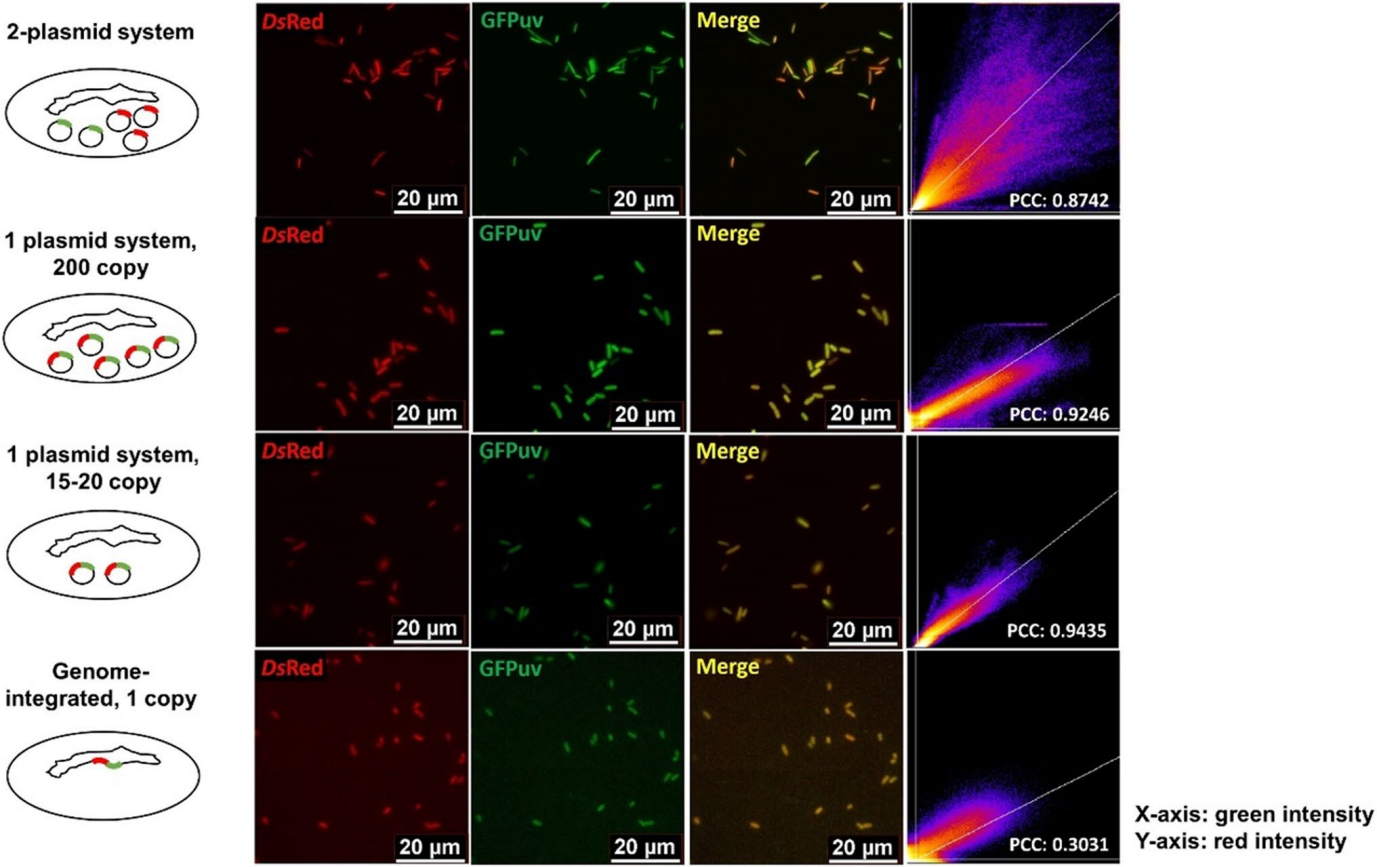
Genome-integrated expression of *Ds*Red and GFPuv showed a more colocalization of the two fluorescent proteins than a multi-plasmid system. Columns from left to right: schematics of different expression systems; Fluorescence imaging in red, green, and merged channels; Scatter plots of green versus red fluorescence. PCC, Pearson’s correlation coefficient

For fluorescence imaging, the genes *DsRed* and *GFPuv* were amplified from existing plasmids and ligated with the PCR-linearized pBluescript II KS+ and pCDF2 backbones, respectively, using the Gibson cloning method (Gibson et al. 2009). *Ds*Red and GFPuv were expressed under the constitutive T7 promoter and the IPTG-inducible P_tac_ promoter, respectively. MG1655(DE3) was co-transformed with pBluescript II KS+_P_T7_-*DsRed* and pCDF2_P_tac_*-GFPuv* to create the strain with two plasmids. To construct the high copy plasmid pBluescript II KS+_P_T7_-*DsRed.GFPuv*, *DsRed* and *GFPuv* were amplified from existing plasmids and assembled as an operon under the constitutive T7 promoter into the PCR-linearized pBluescript II KS+ backbone using Gibson. The plasmid used for the genome integration of *D*sRed and *GFPuv* was created in two steps. First, pTargetF (Jiang et al. 2015) was modified to target the *araA* locus by amplifying the pTargetF backbone using 5′ phosphorylated primers containing a 20-nt sgRNA sequence homologous to the *araA* coding region to be inserted downstream of the J23119 promoter. The PCR-linearized plasmid was then circularized by the T4 ligase (NEB, Ipswich, MA, USA) to create pTargetF_*araA*. Next, the ~500 bp 5′ and 3′ homology regions of the *araA* locus were amplified from the MG1655(DE3) genome, and the operon containing *DsRed* and *GFPuv* was amplified from pBluescript II KS+_P_T7_-*DsRed.GFPuv*. These three gene fragments were cloned into the PCR-linearized pTargetF-*araA* using the Gibson cloning method to create pTargetF_*araA::*P_T7_*-DsRed.GFPuv*. The wildtype MG1655(DE3) cells were transformed with the plasmid pTargetF_ *araA::*P_T7_-*DsRed.GFPuv* to create the single, medium copy plasmid system (Fig. 1). Genome integration was carried out as described in the next section, “genome integration.”

A modified Golden Gate cloning method (Engler et al. 2008) was used to clone the MEP pathway genes. The plasmid pTargetF_*araA* was made Golden Gate-compatible by linearizing it using 5′ phosphorylated primers containing BsaI-HFv2 sites and then re-circularizing by ligation to create the pTargetF_*araA* GG. Plasmids pTargetF_*araA::*P_103/105/106/101/100_*-SIDF* constructed expressed *lacI*, an Anderson promoter with a lac operator, *dxs*, *idi*, *ispDF*, *NAT*^*R*^, and the 5′ and 3′ homology regions of the *araA* locus. All inserts were amplified using primers containing BsaI-HFv2 and Esp3I sites. Gene inserts were first assembled into the part plasmid pYTK001 using Esp3I (NEB, Ipswich, MA, USA) unless mentioned otherwise (Lee et al. 2015). Any BsaI-HFv2 and/or Esp3I sites in the inserts were domesticated prior to assembly (Mukherjee et al. 2021). *lacI* was amplified from the *E. coli* genome MG1655(DE3) using primers containing the constitutive T7 promoter and BsaI-HFv2 sites and cloned into pYTK001. The IPTG-inducible Anderson and T7-lacO promoters were introduced into the vector assemblies as linear, double-stranded oligos containing BsaI-HFv2, and Esp3I sites. Genes including *dxs*, *idi*, and *ispDF* were amplified from the *E. coli* genome and cloned into pYTK001. The synthetic terminator, L3S2P21 (Chen et al. 2013), was linked to the last gene, *ispDF*, in the operon by including the sequence in the reverse primer. The nourseothricin-resistant gene, *NAT*^*R*^, superfolder green fluorescent protein-encoding gene (*sfGFP*), kanamycin-resistant gene (*kan*^*R*^), and the P_tac_ promoter were amplified from existing plasmids and cloned into pYTK001. The components of the integration plasmids pTargetF_*araA::*P_103/105/106/101/100_*-SIDF* were sequentially assembled into pTargetF_araA GG using BsaI-HFv2 (NEB, Ipswich, MA, USA) digestion in four steps. First, the *araA* 5′ homology region, *sfGFP*, and the *araA* 3′ homology region were assembled into pTargetF_*araA GG* to create pTargetF_*araA int GG1*. Next, the *sfGFP* was replaced by *kan*^*R*^, *ispDF*, and *NAT*^*R*^ to create pTargetF_*araA int GG2*. Next, the *kan*^*R*^ was replaced by *sfGFP*, *dxs*, and *idi* to create pTargetF_*araA int GG3*. Finally, the *sfGFP* was replaced by the *lacI* transcription unit and an inducible Anderson promoter to create the final plasmids pTargetF_*araA::*P_103/105/106/101/100_*-SIDF* for genome integration. All plasmids for TargetF_*pykF::*P_103/105/106/101/100/tac/T7-lacO_*-RGHE* containing the 5′ and 3′ homology regions of *pykF*, a promoter, *dxr*, *ispG*, *ispH*, and *ispE*, were cloned and assembled similarly. The native terminator, T_PheA_ (Chen et al. 2013), was placed after *ispE* by including the sequence in the primer. The reverse primers for amplifying the 5′ homology regions of both the *araA* and *pykF* loci included a stop codon to prevent the translation of any non-functional proteins. The sgRNA and homology region sequences were listed in Tables S5 and S6, respectively. The 4-nt overhang sequences used for Golden Gate assembly were listed in the SI Appendix I.

To create the plasmid pCDF2_P_tac_-*ispA(S80F).tObGES* encoding the downstream genes to synthesize geraniol, a mutated *ispA* gene, *ispA(S80F)* or ispA* (GenBank accession #: OR123875), was synthesized from Genewiz (South Plainfield, NJ, USA) and cloned into an existing plasmid containing an N-terminal truncated geraniol synthase from *Ocimum basilicum* (Iijima et al. 2004) and codon-optimized for expression in *E.* coli (GenBank accession #: OR123874).

For the three-plasmid-based system for geraniol production, pTargetF_*araA::*P_106_*-SIDF* (pMB1 origin of replication, ori, spectinomycin resistance, Sp^R^) and pCDF2_P_tac_-*ispA(S80F).tObGES* (200-copy *CloDF1*3 ori, Sp^R^) were modified to encode different antibiotic markers and/or origins of replication to be compatible with pTargetF_*pykF::*P_tac_*-RGHE* (pMB1 ori, Sp^R^). The antibiotic marker and origin of pTargetF_ *araA::*P_106_*-SIDF* were changed to chloramphenicol-resistant gene (*cml*) and *p15A* origin, respectively, to create pIB_*araA::*P_106_*-SIDF*. To construct a plasmid expressing IspA(S80F) *and* t*Ob*GES and compatible with pIB_*araA::*P_106_*-SIDF* and pTargetF_*pykF::*P_tac_*-RGHE*, the pCDFDuet1 plasmid (*CloDF13* ori and Sp^R^) was modified to contain a kanamycin-resistant gene (*kan*^*R*^) instead of the Sp^R^ to create the pCDFDuet1-kan^R^. The PCR-amplified *ispA(S80F)* and *tObGES* were then assembled with the restriction-digested pCDFDuet1-kan^R^ (BamHI and XhoI) (NEB, Ipswich, MA, USA) using the Gibson method to create the pCDFDuet1-kan^R^_P_T7-lacO_-*ispA(S80F)*.*tObGES*. The MG1655(DE3) strain was sequentially transformed with the pIB_*araA::*P_10*6*_*-SIDF*, the pTargetF_*pykF::*P_ta*c*_*-RGHE*, and the pCDFDuet1-kan^R^_P_T7-lacO_-*ispA(S80F)*.*tObGES* to create the plasmid-based strain for geraniol production. All genes inserted into the cloned plasmids were verified by Sanger sequencing (Genewiz, South Plainfield, NJ).

### Genome integration

MG1655(DE3) was sequentially transformed with the pKD46_*Cas9.RecA.Cure* containing *SpCas9*, *recA*, and the genes encoding λ Red recombinases, and the pTargetF_*araA::*P_103/105/106/101/100_*-SIDF* bearing the gRNA and the MEP pathway genes to be inserted under different promoters and flanked by ~500 bp 3′ and 5′ homologous regions of *araA* locus. Cells were plated on the LB medium containing 50 μg/ml carbenicillin for pKD46_*Cas9.RecA.Cure*, 100 μg/ml spectinomycin for pTargetF_ *araA::*P_103/105/106/101/100_*-SIDF*, and 0.5% (w/v) arabinose (Acros Organics, Geel, Belgium) to induce the expression of the λ Red recombinases and grown at 30 °C for 24 h. Colonies were screened by PCR for the expected genome integration. The pTargetF_P_103/105/106/101/100_*-SIDF* plasmids were cured by streaking out the colony with the intended genome integration on LB plus carbenicillin but with no spectinomycin at 30 °C overnight. The loss of the pTargetF_P_103/105/106/101/100_-SIDF plasmids was validated by no growth on an LB plus spectinomycin plate at 30 °C overnight. The resulting strain with the *araA* integration and the pKD46_*Cas9.RecA.Cure* plasmid was then transformed with pTargetF_*pykF::*P_103/105/106/101/100/tac/T7-lacO_*-RGHE* plasmids individually and grown on an LB plate with carbenicillin, spectinomycin, and arabinose to initiate the integration at the *pykF* locus at 30 °C for 24 h. The resulting colonies were screened by PCR for the expected genome integration. The pTargetF_P_103/105/106/101/100/tac/T7-lacO_*-RGHE* and pKD46_*Cas9.RecA.Cure* plasmids were cured simultaneously by growing the colony with the correct integration on a plain LB plant at 37 °C overnight due to the temperature-sensitive origin of replication, *repA101ts*, of pKD46_*Cas9.RecA.Cure* which degrades at 37 °C. The absence of both plasmids was verified by streaking cells on LB plates containing either carbenicillin or spectinomycin.

### Fluorescence imaging

Samples for imaging were cultured at 16 °C and harvested 16 h after induction. Cells of OD_600_ ~3 were centrifuged at 17,000 rpm for 2 min. The supernatants were discarded, and the pellets were resuspended in 100 μL of 0.25% (w/v) sterile melt agarose (Bio-Rad, Hercules, CA, USA). Five microliters of the samples were spotted on slides, covered with coverslips, and sealed around the edges of the coverslips using clear nail polish before imaging.

Cells were visualized on a Leica DMi 8 inverted microscope equipped with an sCMOS Leica camera (Wetzlar, Germany) using a 63× oil immersion objective. The excitation/emission wavelengths used for *Ds*Red and GFPuv were 558/583 nm and 405/510 nm, respectively. Images were individually thresholded in Fiji (ImageJ) (Schindelin et al. 2012) to minimize background fluorescence. Scatter plots were constructed using the Colocalization Finder plugin. Pearson’s correlation coefficients were calculated using the Just Another Colocalization Plugin (JACoP) (Bolte and Cordelières 2006).

### Quantitative real-time polymerase chain reaction (qRT-PCR)

qRT-PCR was used to quantify the gene expression levels in the MEP pathway under promotors of different strengths. Cells from the wildtype strain and engineered strains with relatively weak (*araA::*P_103_*-SIDF/pykF::*P_103_*-RGHE*), medium (*araA::*P_106_*-SIDF/pykF::*P_106_*-RGHE*), and strong (*araA::*P_100_*-SIDF/pykF::*P_100_*-RGHE*) promotors were cultured in biological triplicates at 30 °C post induction for 5.5 h as described in “culturing conditions” above. One milliliter culture from each flask was harvested for RNA extraction using the TRIzol^TM^ reagent (Life Technologies, Carlsbad, CA, USA) following the manufacturer’s protocol. Three hundred and fifty nanograms of extracted RNA samples were then converted to cDNA using the iScript™ cDNA synthesis kit (Bio-Rad, Hercules, CA, USA) using the following thermo cycles: priming for 5 mins at 25 °C, reverse transcription for 20 min at 46 °C, and reverse transcriptase inactivation for 1 min at 95 °C, in a total reaction volume of 15 μL.

Primers for qRT-PCR were designed for each MEP pathway gene (Table S7). qRT-PCR was conducted using *idnT* (Zhou et al. 2011) as the internal reference gene. Primers were validated using 10^1^- to 10^5^-fold dilutions of cDNA samples. The 10 μL qPCR reaction mix was composed of respective primer pairs (final concentration 400 nM), 2 μL ten-fold diluted cDNA as the template, sterile double distilled water, and 5 μL 2X Universal SYBR Green Fast qPCR Mix (ABclonal Technology, Woburn, MA, USA), according to the manufacturer’s protocol. The reaction ran under the following thermo cycless: initial denaturation for 3 min at 95 °C, followed by 40 cycles of denaturation for 10 s at 95 °C, and annealing for 30 s for 55 °C. In a final melting step, melting curves were generated from 55 to 95 °C in 0.5 °C increments for 81 cycles. Two technical replicates and three biological replicates were used to quantify gene expression from promoters of varying strength and the wildtype strain.

### Gas chromatography coupled with mass spectrometry (GC/MS) for quantifying geraniol

Samples were harvested 36 h post induction. For each sample, 500 μL culture was centrifuged in a 1.5 mL microfuge tube at 17,000 rpm for 2 min. The supernatant was collected in another 1.5 mL microfuge tube to which 500 μL hexane was added. The tube was vortexed vigorously for 5 min to extract the geraniol into the hexane phase and then spun down at 17,000 rpm for 2 min. Four hundred microliters of the hexane phase containing geraniol were transferred into a glass sample vial and stored at −20 °C until analysis.

A Thermo Trace 1310 gas chromatograph with a Thermo Scientific TraceGOLD TG-5SILMS column (30 m long, 0.25 mm inner diameter, 0.25 μm film thickness) (Thermo Fisher Scientific, Waltham, MA, USA) was used to separate the geraniol in the samples. Helium was used as the carrier gas at a flow rate of 1 mL/min. The injector was held at 200 °C, and the column was injected with 5 μL of each sample using the TriPlus RSH Autosampler. The oven was initially held at 40 °C for 4 min, then ramped up to 280 °C at the rate of 20 °C/min, and finally held at 280 °C for 2 min. A Thermo Q-exactive^TM^ Orbitrap Tandem mass spectrometer (Thermo Fisher Scientific, Waltham, MA, USA) was used to detect geraniol. A mass range of 39–200 m/z was monitored in the positive ion mode. Geraniol eluted at 10.24 min. Geraniol was quantified using Xcalibur^TM^ (Thermo Fisher Scientific, Waltham, MA, USA) based on a standard curve built using 1.5 – 25 mg/L pure geraniol (Acros Organics, Geel, Belgium) in hexane. Peak areas of the ion of m/z 123.1168 ± 5 ppm were measured.

### Liquid chromatography coupled with mass spectrometry (LC/MS) for quantifying MEP pathway intermediates

Samples were harvested 5.5 h post induction, in the mid to late-log phase. Samples were prepared as described in a previous study (González-Cabanelas et al. 2016). Ten milliliters of culture were collected for each sample. After centrifuging at 20,000 × *g* for 15 min, the supernatant was discarded, and the pellet was resuspended in 1 mL methanol:acetonitrile:water (2:2:1) containing 0.1% ammonium hydroxide. After incubating on ice for 15 min and centrifuging the cells at 20,000 × *g* for 5 min, the supernatant containing the intracellular metabolites was extracted and dried under a stream of nitrogen in a 40 °C heat block. The dried residue was dissolved in 100 μL of 10 mM ammonium acetate (adjusted to pH=9 with ammonium hydroxide) and centrifuged at 20,000 × *g* for 5 min. Ninety microliters of supernatant were collected in a fresh 1.5-ml microfuge tube and centrifuged for 5 min at 20,000 × *g* to remove any residual particles. Eighty microliters of supernatant were collected in a glass sample vial for LC/MS immediately without storage.

A Thermo UltiMate 3000 ultra-high performance liquid chromatography system fitted with an Atlantis Premier BEH Z-HILIC VanGuard FIT column (1.7 μm particle size, 2.1 mm long, 100 mm inner diameter) (Thermo Fisher Scientific, Waltham, MA, USA) to separate the MEP pathway metabolites. The LC column compartment was maintained at 25 °C. Ten microliters of each sample were injected into the column. Solution A was 50 mM ammonium carbonate in water, and solution B was acetonitrile. Isocratic elution was performed at 70% solution B. The flow rate was 0.2 mL/min. A Q-exactive Focus Orbitrap tandem mass spectrometer (Thermo Fisher Scientific, Waltham, MA. USA) was used to detect the metabolites. Electrospray ionization was conducted in the negative-ion mode. The MS scan range was 50–600 m/z at a resolution of 70,000. The metabolites were quantified using Xcalibur^TM^ based on standard curves built using 0.49 – 62.5 mg/L 1-deoxy-D-xylulose 5-phosphate (DXP), 0.49 – 125 mg/L 2-methyl-D-erythritol 4-*phosphate* (MEP), 0.97 – 250 mg/L 4-diphosphocytidyl-2-methylerythritol (CDP-ME), 1.95 – 1000 mg/L 2-methyl-D-erythritol-2,4-cyclopyrophosphate (MEcPP), 0.97 – 125 mg (E)-4-hydroxy-3-methyl-but-2-enyl pyrophosphate (HMBPP) (Echelon Biosciences, Inc., Salt Lake City, UT, USA), and 0.97 – 1000 mg/L dimethylallyl pyrophosphate (DMAPP) (Cayman Chemical Company, Ann Arbor, MI, USA) commercial standards prepared in 10 mM ammonium acetate were adjusted to pH=9 using ammonium hydroxide. Peak areas of the [M-H]^-^ ions of m/z 213.017 ± 5 ppm, 215.0326 ± 5 ppm, 520.0739 ± 5 ppm, 276.9884 ± 5 ppm, 260.9935 ± 5 ppm, and 244.9986 ± 5 ppm were measured for DXP, MEP, CDP-ME, MEcPP, HMBPP, and DMAPP respectively.

### Genetic stability

Geraniol-producing cell cultures were harvested 36 h, 60 h, and 84 h post induction. A hundred microliters of 10^4^ – 10^8^ ten-fold serial dilutions were plated on non-selective LB agar and selective LB agar containing nourseothricin for the genome-integrated strain or chloramphenicol and spectinomycin for the three-plasmid strain. After incubating at 37 °C overnight, the number of colonies on each plate was counted. The colony forming unit (CFU) of each culture was calculated by multiplying the number of colonies on the respective plates with the respective dilution factors.

### Principal component analysis

Principal component analysis (PCA) was performed using packages “devtools” and “gridExtra” in R language (Chambers 2008). The metabolic data were mean-centered and scaled to unit variance, which was calculated by subtracting each metabolite value from the mean and dividing it by the standard deviation. The complete code was listed in the “Supplemental codes” section of the supplemental information.

## Results

### Decreasing gene copy numbers resulted in uniform gene expression

Metabolic pathways are often overexpressed from one or more plasmids in *E. coli* (Liu et al. 2016; Wang et al. 2021; Yang and Guo 2014). However, production titers are influenced by plasmid copy numbers (Ajikumar et al. 2010; Birnbaum and Bailey 1991; Silva et al. 2012). To demonstrate how plasmid-based overexpression affects pathway productivity, we created four *E. coli* strains: (1) a strain separately expressing two fluorescent proteins, *Ds*Red and GFPuv, from two different plasmids; (2) a strain expressing *DsRed* and *GFPuv* as an operon on a single high-copy plasmid (200 copies); (3) a strain expressing these two genes from one medium-copy plasmid (15–20 copies); and (4) a strain with the two genes integrated into the genome as a single operon (one copy). Fluorescence imaging showed that cells expressing *Ds*Red and GFPuv from two separate plasmids exhibited different colors, including red, green, and yellow, indicating that the genes were expressed at various levels in different cells (Fig. 1). The scatter plots and Pearson’s correlation coefficients (PCC) showed the green and the red fluorescence vary significantly in different cells. Cells expressing the two proteins from the same plasmid or genome integration exhibited yellow colors with different intensities, indicating both proteins were expressed uniformly in each cell. The differences in color intensities reflected the differences in gene copy numbers. The lower PCC value for the genome-integrated strain was likely due to the low signal from the single copy of the genes and a high background fluorescence. Although both the single plasmid systems and the genome-integrated system resulted in a uniform expression, genome-integration was preferable for overexpressing long metabolic pathways such as the eight-gene MEP pathway because of the additional genetic stability and the limitation of cloning and transforming a large plasmid.

### Genome-integration of the complete MEP pathway

**Fig. 2:**
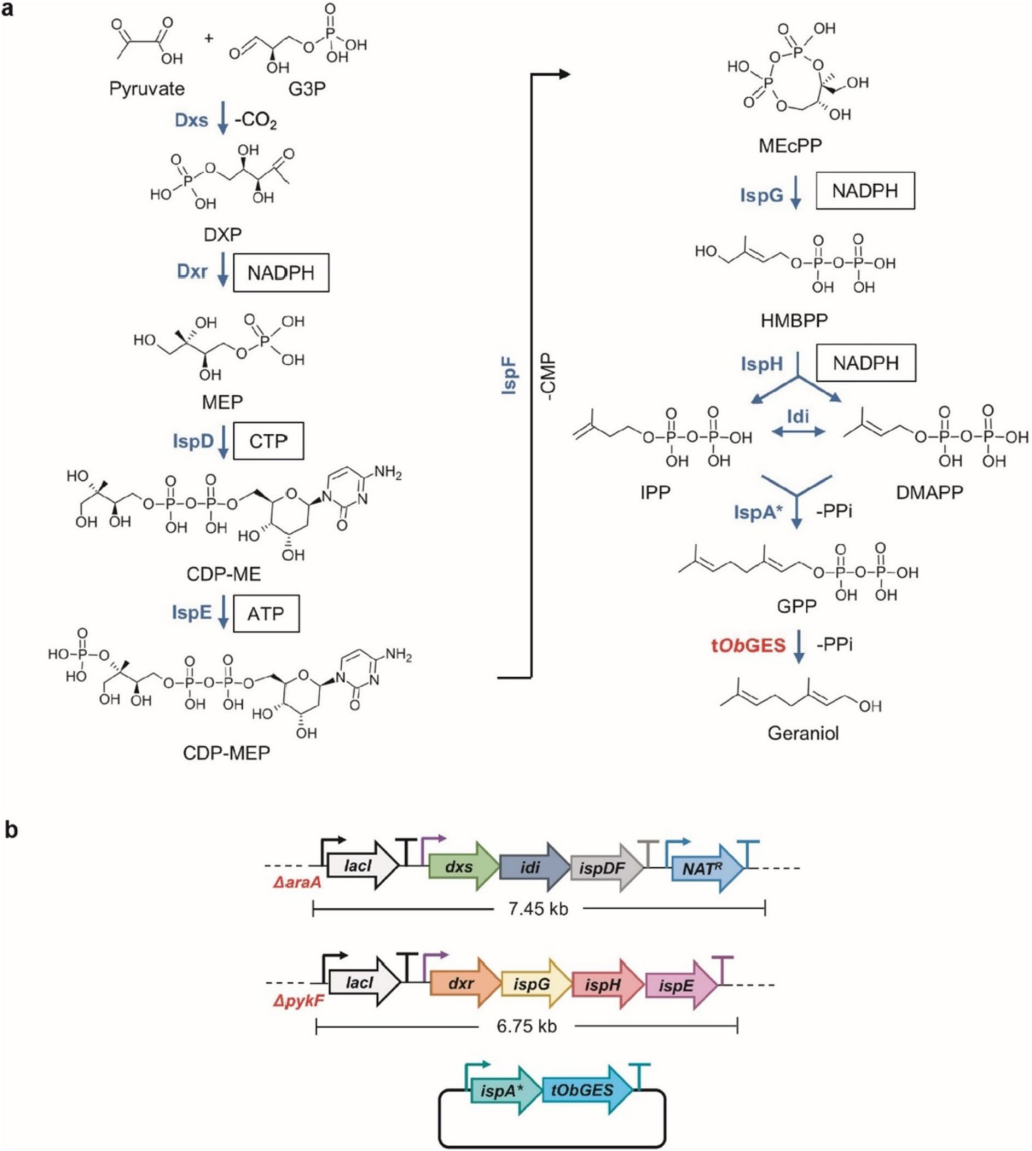
Integrating an additional copy of the MEP pathway into the *E. coli* genome for geraniol production. **a** A schematic of the MEP pathway. The pathway enzymes were shown in blue. Dxs, 1-Deoxy-D-xylulose 5-phosphate (DXP) synthase; Dxr, 1-Deoxy-D-xylulose 5-phosphate (DXP) reductoisomerase; IspD, 2-C-Methyl-D-erythritol 4-phosphate (MEP) cytidylyltransferase; IspE, 4-Diphosphocytidyl-2-C-methylerythritol (CDP-ME) kinase; IspF, 2-C-Methyl-D-erythritol-2,4-cyclopyrophosphate (MEcPP) synthase; IspG, (E)-4-Hydroxy-3-methyl-but-2-enyl pyrophosphate (HMBPP) synthase; IspH, (E)-4-Hydroxy-3-methyl-but-2-enyl pyrophosphate (HMBPP) reductase; Idi: Isopentenyl-diphosphate (IPP) delta isomerase 1; IspA*, *E. coli* IspA (S80F) mutant acting as a geranyl pyrophosphate synthase; t*Ob*GES: N-terminal truncated geraniol synthase from *Ocimum basilicum*. Boxed cofactors were consumed in the respective steps. NADPH, nicotinamide adenine dinucleotide phosphate; CTP, cytidine triphosphate; ATP, adenosine triphosphate; CMP, cytidine monophosphate; PPi, pyrophosphate. **b**. Schematic of the genome-integration of the MEP pathway genes. IspA* and t*Ob*GES were expressed from a plasmid. Pathway genes were expressed from various inducible Anderson promoters. LacI and Nat^R^ were expressed by constitutive promoters. IspA* and t*Ob*GES were expressed under the IPTG-inducible P_tac_ promoter. The sizes of both genome-integrated cassettes were shown

We overexpressed the complete *E. coli* MEP pathway (Fig. 2a) by integrating an additional copy of the eight MEP genes into the genomic loci *araA* and *pykF*, respectively. The gene *araA* encoded L-arabinose isomerase, whose disruption prevented the breakdown of exogenous arabinose necessary to induce the expression of the λ phage’s recombinase during genome editing. The gene *pykF* encodes pyruvate kinase, whose knockout increased the MEP pathway flux by balancing pyruvate and glyceraldehyde-3-phosphate (G3P), the two precursors of the MEP pathway (Al Zaid Siddiquee et al. 2004; Farmer and Liao 2001; Li et al. 2015). The MEP pathway genes, including *dxs*, *idi*, and *ispDF,* which were previously reported as rate-limiting (Yuan et al. 2006), were inserted into the *araA* locus (denoted as *araA*::*SIDF*), whereas *dxr*, *ispG*, *ispH*, and *ispE,* were inserted into the *pykF* locus (denoted as *pykF*::*RGHE*) as operons using the CRISPR/Cas9 guided gene editing (Fig. 2b) (Jiang et al. 2015). The genome-integrated pathway was expressed under the IPTG-inducible promoters, and the *lac* repressor LacI was overexpressed constitutively to suppress the leaky expression from the IPTG-inducible promoters. In addition, a nourseothricin-resistant marker expressed from its native promoter was included in the *araA* locus for authentication. The engineered *E. coli* strain having the genomically integrated and overexpressed MEP pathway was transformed with a plasmid expressing IspA*, encoding an engineered geraniol pyrophosphate synthase, and t*Ob*GES, a geraniol synthase from *Ocimum basilicum* truncating the targeting peptide, to enable the synthesis of geraniol, which is a valuable monoterpene alcohol and a precursor of the medicinally important monoterpene indole alkaloids (Brown et al. 2015; Chen and Viljoen 2010; Reiling et al. 2004).

### Genome integration afforded a higher geraniol titer compared to the plasmid-based expression

**Fig. 3:**
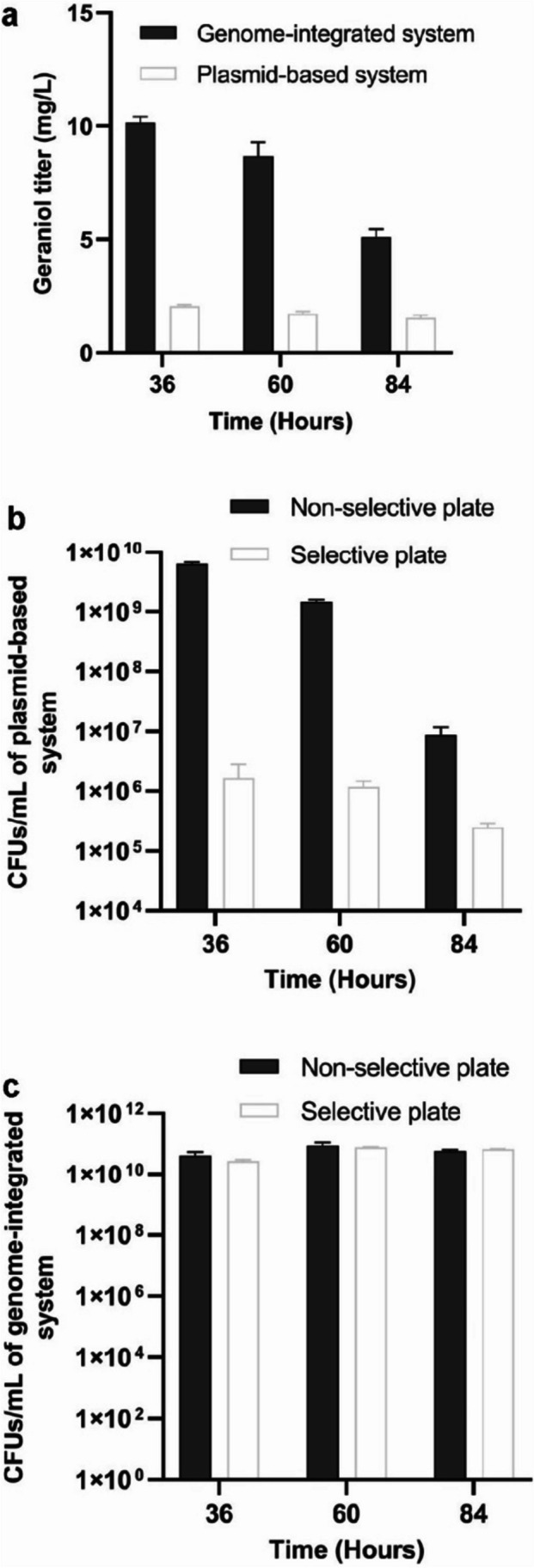
Genome-integration of the MEP pathway resulted in higher geraniol production and genetic stability. **a** Geraniol production from the genome-integrated and the plasmid-based strains over 3 days post inoculation. Data represent the average ± S.D. of three biological replicates. **b** Number of colony forming units (CFU) per milliliter culture of the plasmid-based strain over 3 days. Cells were plated on non-selective plates and selective plates containing chloramphenicol and spectinomycin to select the two MEP-gene-containing plasmids. **c** CFU per milliliter culture of the genome-integrated strain over 3 days. Cells were plated on non-selective plates and selective plates with nourseothricin. Cultures were diluted 10^4^~10^8^ folds and plated in triplicates. Error bars represent the average ± S.D. of three independent biological replicates

To compare the geraniol productivity from the genome-integrated system and a plasmid-based system, we created a strain overexpressing MEP genes from two plasmids, pIB_*araA::*P_106_*-SIDF* and pTargetF_*pykF::*P_tac_*-RGHE*, respectively. The genome-integrated strain with the same promoters, *araA::*P_106_*-SIDF/ pykF::*P_tac_*-RGHE*, produced five-fold more geraniol than the plasmid-based strain suggesting that the non-uniform gene expression from plasmids might hamper the product titer (Fig. 3a). To determine if the genome-integrated strain was genetically more stable, we plated the liquid cultures onto solid plates with or without the antibiotics each day during the cultivation. The plasmid strain showed over 1,000-fold more colonies on the non-selective plates compared to the selective plates. This difference reflected the overwhelming loss of one or both plasmids in the plasmid-based strain, despite the antibiotics in the liquid culture. We also observed a greater loss of the pIB_*araA::*P_106_*-SIDF* plasmid compared to the pTargetF_*pykF::*P_tac_*-RGHE* plasmid, presumably due to the lower copy number of the pIB_*araA::*P_106_*-SIDF* plasmid (Fig. S1). Cells that lost plasmids died over time due to the antibiotics in the liquid culture. The cell death was evidenced by the sharply decreased cell numbers on the non-selective plate over time (Fig. 3b). The genome-integrated strain had similar numbers of colonies on the non-selective and selective plates at all times (Fig. 3c). The cell viability of the genome-integrated strain was maintained over the course of the cultivation. The genetic instability of the plasmid-based strain was likely responsible for the decreased geraniol production compared to the genome-integrated strain.

### Constructing a strain library to increase geraniol production and quantify MEP pathway intermediates

**Fig. 4:**
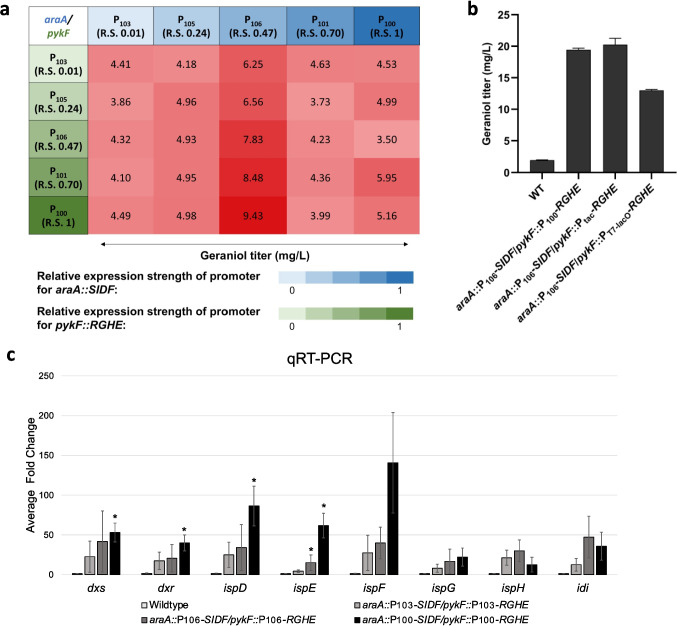
Modulating the MEP pathway gene expression to increase geraniol production. **a** Geraniol production from a combinatorial strain library expressing the heterologous MEP pathway genes from different IPTG-inducible Anderson promoters (iGEM Registry: BBa_K1585103, BBa_K1585105, BBa_K1585106, BBa_K1585101, BBa_K1585100). Promoters expressing the *araA::SIDF* and the *pykF::RGHE* operons were shown with increasing intensities of blue and green, respectively, reflecting promoter strengths. The relative promoter strengths (R.S.) ranged from 0.01 to 1, were listed in the iGEM Registry (http://parts.igem.org/Promoters/Catalog/Anderson). Geraniol titers (mg/L) were shown with different intensities of crimson. **b** Geraniol production from additional strains with IPTG-inducible P_tac_ and T7-lacO promoters for *pykF::RGHE*. Cells were harvested 36 h post induction. **c** qRT-PCR analysis quantifying the expression of MEP pathway genes in three representative strains in the combinatorial strain library. Asterisks denote *p*<0.05 in one-tailed Student’s *t* test compared with the wildtype. WT, wildtype. Strain genotypes are listed in Table S1. Error bars represent the average ± S.D. of three independent biological replicates

To optimize the MEP pathway genes’ expression, we created a 27-strain library by combinatorially testing different promoters for the integrated MEP pathway genes. IPTG-inducible Anderson promoters weaker than the P_trc_ and the T7-lacO promoters were used to avoid the metabolic burden and to titrate the promoter strengths (Balzer et al. 2013; Noh et al. 2017). The MEP pathway genes, including *dxs*, *idi*, and *ispDF*, which were previously reported as rate-limiting, were inserted into the *araA* locus (denoted as *araA::SIDF*), whereas *dxr*, *ispG*, *ispH*, and *ispE*, were inserted into the *pykF* locus (denoted as *pykF::RGHE*) as operons using the CRISPR/Cas9 guided gene editing (Fig. 2b). Interestingly, when the promoter strength of the *araA::SIDF* increased, the geraniol titer increased first but then declined. The strains with the medium strength promoter K1585106 (P_106_) for *araA::SIDF* showed the highest geraniol production when the promoter of the *pykF::RGHE* was held constant. This observation suggested that at least one of the *araA::SIDF* enzymes, including Dxs, Idi, IspD, and IspF, was regulated beyond the transcriptional level, perhaps under feedback inhibition. When the *araA::SIDF* was under the medium-strength P_106_ promoter, the geraniol titer increased as the promoter strength of *pykF::RGHE* increased (Fig. 4a). We verified if the titer would further increase by further increasing the promoter strength of *pykF::RGHE* using stronger IPTG-inducible promoters such as P_tac_ and T7-lacO. However, using the P_tac_ promoter for *pykF::RGHE* resulted in a similar level of geraniol, whereas the T7-lacO promoter decreased geraniol production, likely due to regulations downstream of transcription (Fig. 4b). Quantifying the expression level for each gene in the MEP pathway using qRT-PCR revealed that the gene expression corresponded well with the expected promoter strengths for all the genes except *ispH* and *idi*, for which the differences among the three strains were not statistically significant (Fig. 4c). No obvious growth defect was observed in all engineered strains since they all had similar OD_600_ readings (Fig. S2).

**Fig. 5:**
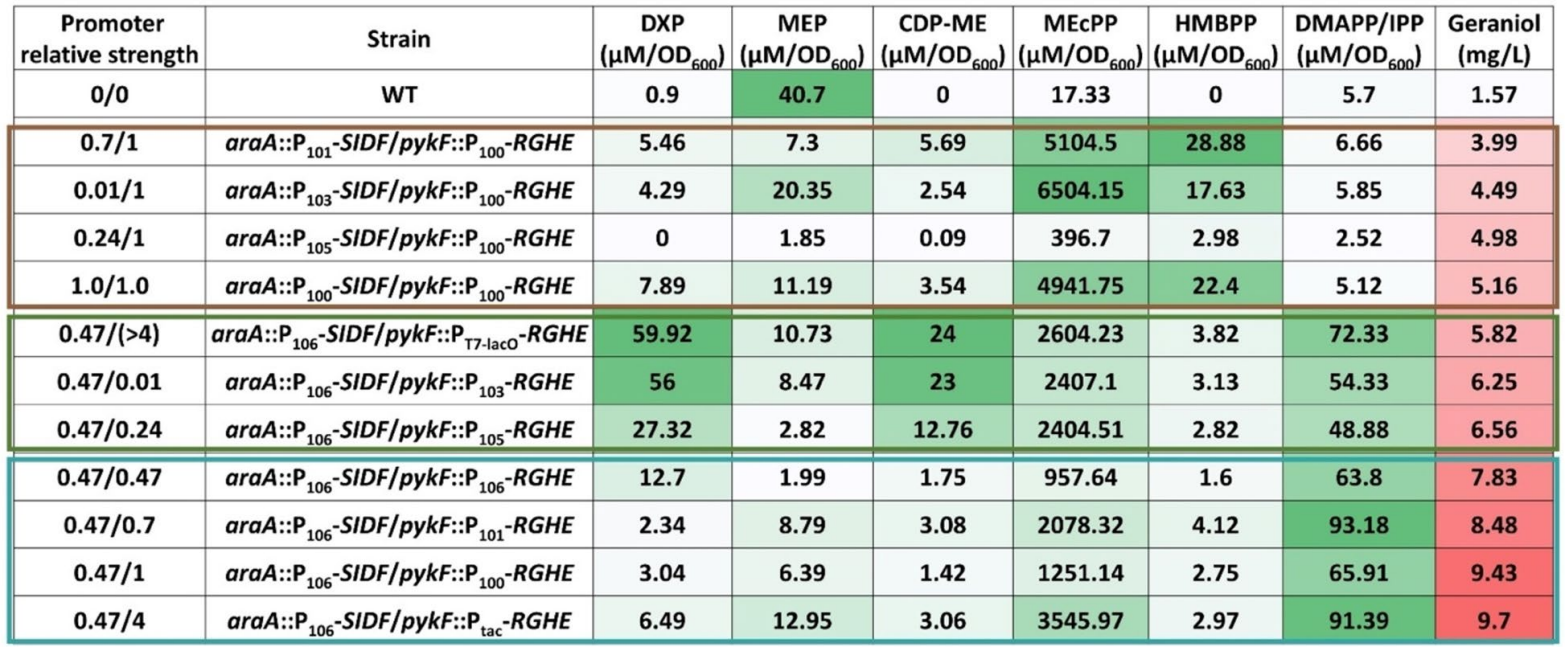
Quantifying MEP pathway intermediates in engineered *E. coli* strains. The intracellular metabolite concentrations were shown with increasing intensities in green. IPP and DMAPP were isomers that coeluted. The geraniol titers were shown with increasing intensities in crimson. The numbers under “Promoter relative strength” indicated the relative strengths of promoters. The strains boxed in brown, green, and cyan were the “low producers,” “medium producers,” and “top producers,” respectively. All quantifications were performed with biological triplicates

Because results from the combinatorial library suggested regulations beyond the transcription level, we quantified the intracellular concentrations of all MEP pathway intermediates except 4-diphosphocytidyl-2-C-methylerythritol 2-phosphate (CDP-MEP), an unstable intermediate (Li and Sharkey 2013), in 11 of the 27 strains in the combinatorial library, representing a broad range of geraniol titers (Fig. 5). The quantification was performed during the cells’ exponential growth when the intermediate concentrations were stable and high. It was immediately noticeable that strains having high concentrations of IPP/DMAPP, the universal five-carbon terpene precursors, had medium to high geraniol titers. However, relationships between other intermediate concentrations and the geraniol titer were not obvious, thus warranting an in-depth statistical analysis.

### Principal component analysis revealed how MEP pathway intermediates correlate with geraniol production

**Fig. 6:**
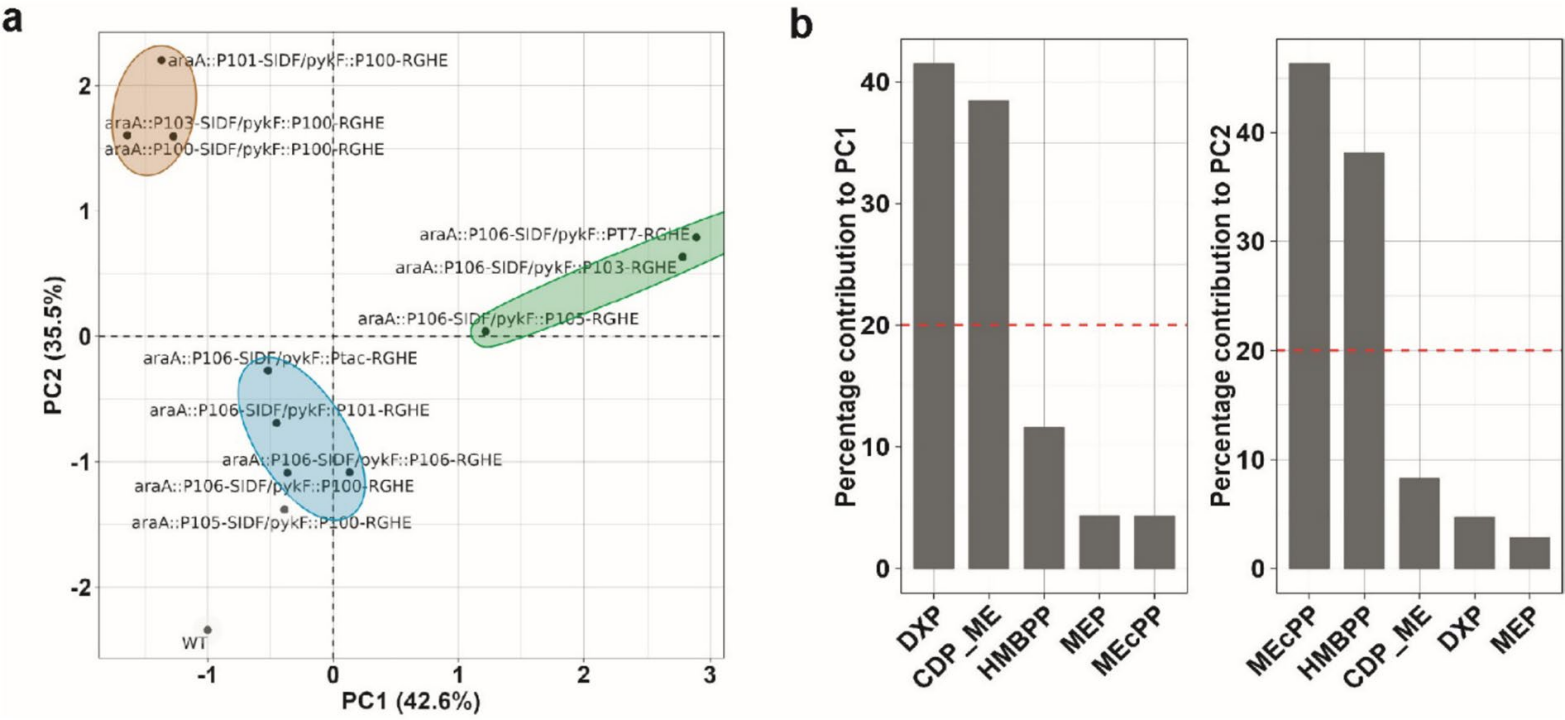
Principal component analysis (PCA) of intracellular MEP pathway intermediates except IPP/DMAPP in the engineered *E. coli* strains. **a** Biplot of principal components (PC) 1 and 2 separating “top producers” (cyan ellipse), “medium producers” (green ellipse), and “low producers” (brown ellipse). The circled strains corresponded with the boxed strains of the same respective colors in Fig. 5. WT, wildtype strain. **b** Percentage contributions of each quantified metabolite to PC1 and PC2. The dashed line indicates the expected average contribution of five variables, which was 20%, the cutoff of importance

To determine the contribution of other MEP pathway intermediates to geraniol titer, we performed a principal component analysis (PCA) using the metabolic data gathered in Fig. 5. The strains formed three distinct clusters along the principal component 1 (PC1) and principal component 2 (PC2) axes correlating to the geraniol titers (Fig. 6a). These three clusters were the top-producers, the medium-producers, and the low-producer clusters. The top producers clustered towards the lower middle part of the PCA space, while the medium producers clustered to the middle right, and the low producers were at the top left of the plot. Further analysis of the constituents of PC1 and PC2 revealed that DXP and CDP-ME were the major contributors to PC1, and MEcPP and HMBPP were the major contributors to PC2 (Fig. 6b). These results demonstrated that moderate levels of DXP and CDP-ME coupled with medium-low levels of MEcPP and HMBPP were required to achieve high titers of geraniol. The geraniol production decreased with increased DXP and CDP-ME in the medium producers and further decreased with the accumulation of MEcPP and HMBPP but diminishing DXP and CDP-ME in the low producers. The wildtype strain lays at the bottom left of the PCA plot due to the low levels of all the intermediates except MEP (Figs. 5 and Fig. 6a). It was clear that a medium DXP level was the most critical criterion to maximize geraniol production. Additionally, MEP contributed the least to PC1 and PC2 and therefore was the least influential intermediate for geraniol production (Fig. 6b). The strain *araA::*P_105_*-SIDF/pykF::*P_100_*-RGHE* was an outlier as it clustered close to the high producers but produced low levels of geraniol (Fig. 5) possibly due to a technical issue during metabolite extraction. We also tried to include IPP/DMAPP with all other pathway intermediates in another PCA analysis. The resulting plot still formed three distinct clusters corresponding to the geraniol titers (Fig. S3a). Although the contributions of each intermediate were less prominent, DXP and CDP_ME were still the top two contributors to PC1, and MEcPP and HMBPP were the primary contributors to PC2 (Fig. S3b).

## Discussion

In this work, we studied the impact of overexpressing the entire MEP pathway through genome integration in *E. coli* on producing monoterpene geraniol. Though the MEP pathway has been targeted for overexpression in *E. coli* in the past, there has been no report on modulating the expression of the entire genome-integrated pathway to maximize terpene titers. Integrating another copy of the MEP pathway into the *E. coli* genome has the advantage of maintaining the native MEP pathway for cell growth and the flexibility to genomically integrate heterologous MEP genes from other species. To construct a genetically stable and balanced strain for terpene production, we created a combinatorial strain library with different promoters for the genome-integrated MEP pathway and used geraniol as the reporter. Quantifying the intracellular pathway intermediate concentrations revealed the optimal levels of these intermediates to maximize the geraniol titer.

The common practice of expressing metabolic genes from multiple plasmids leads to high gene expression but also results in genetic variability and instability. Genomic integration of metabolic pathways maintains genetic stability, but the expression is limited by the gene copy number. Integrating the MVA pathway into the *E. coli* genome significantly decreased isoprenoid productivity compared to a plasmid-based expression system, partially due to the decreased gene copy number (Alonso-Gutierrez et al. 2018). Indeed, increasing the copies of genome-integrated genes by the RecA-mediated chemically induced chromosomal evolution (CIChE) produced isobutanol levels comparable to a plasmid-based expression system in *E. coli* (Saleski et al. 2021; Tyo et al. 2009). In our study, the single-copy, genome-integrated MEP pathway produced significantly more geraniol than the plasmid-based strain, which may partially attribute to the genetic variability among cells and/or dramatic plasmid loss in the plasmid-based expression system (Figs. 1 and 3b). These observations also suggested that a fine balance of pathway intermediates, which is more achievable with genome integration than the plasmid-based system, is essential to increase the flux of a highly regulated pathway such as the MEP pathway.

The highest geraniol production in our strain library resulted from a medium strength promoter for the genes *dxs*, *idi*, and *ispDF* (Fig. 4). This concurred with a previous study in which the highest taxadiene production resulted from relatively weak expression of the same four genes (Ajikumar et al. 2010). Indeed, the PCA analysis (Fig. 6) demonstrated that medium levels of the Dxs and IspD products, DXP, and CDP-ME, respectively, were required to maximize geraniol production. Dxs is the primary rate-limiting step in the MEP pathway since it showed the highest flux control coefficient (Volke et al. 2019; Wright et al. 2014). Our PCA results supported this conclusion as DXP, the product of Dxs, exerted the highest control over terpene production. Dxs, the first enzyme in this pathway, was feedback inhibited by the end products, IPP and DMAPP, typical in metabolic regulations. This allosteric regulation explained why expressing Dxs from a strong promoter decreased terpene titers (Fig. 4a). A recent report delineated how IPP and DMAPP feedback inhibit Dxs:IPP and DMAPP bind to the dimerization surface of Dxs, converting the active dimer into inactive monomers, followed by protein aggregation. This elegant mechanistic elucidation enables rationally designing Dxs to circumvent this key feedback inhibition in the future (Di et al. 2022). Idi interconverts IPP and DMAPP. Although nonessential for normal bacterial growth as IspH synthesizes IPP and DMAPP simultaneously, overexpressing *idi* increased carotenoid titer and is a common strategy in metabolic engineering (Hahn et al. 1999). Idi can optimize the IPP:DMAPP ratio since IspH generates IPP:DMAPP at a fixed 5:1 ratio, whereas the required IPP:DMAPP ratio varies depending on the class of terpene produced (Walsh and Tang 2017).

It is generally understood that intermediates of a pathway must be consumed effectively to prevent flux imbalances (Jones et al. 2015). Based on the metabolic data in Fig. 5, it was tempting to assume that high levels of terpene precursors, IPP and DMAPP, led to high geraniol titers. However, our PCA results showed that IPP and DMAPP levels do not solely determine geraniol production. It was essential to maintain all the intermediates upstream of IPP and DMAPP at medium or medium-low levels while maximizing the IPP and DMAPP concentrations to maximize geraniol production. The medium producers accumulated DXP and CDP-ME, preventing them from reaching high geraniol titers. Furthermore, the low producers accumulated MEcPP and HMBPP (Figs. 5 and 6a). The lack of conversion of MEcPP and HMBPP to their respective products, despite the strong overexpression of IspG and IspH in these strains, suggested that these two enzymes were limited by processes other than transcription. One of these processes could be the limited *E. coli* iron-sulfur maturation system encoded by the *isc* operon. Overexpressing the *isc* operon increased IspG and IspH’s activities by a few orders of magnitude (Gräwert et al. 2004; Zepeck et al. 2005; Zhao et al. 2013b). Thus, overexpressing the *isc* operon in the top producers will further increase terpene production in the future. The PCA analysis also identified MEP, the product of Dxr and the first committed MEP pathway intermediate, had the least influence on terpene titer. This result explained a previous observation that overexpressing Dxr did not increase carotenoid production (Yuan et al. 2006).

To summarize, this study created an *E. coli* platform strain for terpene production by inserting an additional copy of the complete MEP pathway into the genome. The genome-integrated MEP pathway was genetically stable and reached a higher geraniol titer than a plasmid-based system with the same promoters. Modulating the expression of the genome-integrated MEP pathway genes revealed that a delicate balance of the pathway intermediates was critical to maximize terpene titer. In particular, IPP/DMAPP and DXP levels primarily determined final terpene yields. The highest geraniol-producing strains can be used as chassis to synthesize any terpene by transforming a plasmid containing a specific prenyltransferase and a terpene synthase.

## Supplementary information

Supplementary file 1 (PDF 662 kb)

## Data Availability

All data can be provided by the corresponding author upon request for non-commercial usage.
