# Supplementary material for: The non-mevalonate pathway requires a delicate balance of intermediates to maximize terpene production
Source: Appl Microbiol Biotechnol. 2024 Feb 29;108(1):245. doi: 10.1007/s00253-024-13077-7 (PMC10904526; doi:10.1007/s00253-024-13077-7)
Supplement: Supplementary file 1 — (PDF 662 kb) [file 253_2024_13077_MOESM1_ESM.pdf]

Supplemental Information

The Non-Mevalonate Pathway Requires a Delicate Balance of Intermediates to Maximize Terpene Production

Indu Raghavan[1], Rosheena Juman, Zhen Q. Wang*[1]

1. *Department of Biological Sciences, University at Buffalo, the State University of New York, Buffalo, New York, NY14260, United States*

**Corresponding author: Zhen Q. Wang (zhenw@buffalo.edu), 653 Cooke Hall, Department of Biological Sciences, University at Buffalo, Buffalo, NY14260*

Supplemental Methods

Genetic stability assay

The genetic stability assay in Fig.S1 was performed as follows. Cultures were grown in triplicates, and samples from each flask were harvested at 36 h, 60 h, and 84 h post-induction. Cultures were plated on non-selective LB agar and incubated overnight at 37°C. A hundred colonies from each non-selective plate were streaked out on the respective selective media: LB + nourseothricin for the genome-integrated strain, LB + spectinomycin, and LB + chloramphenicol for the plasmid strain to test the presence of additionally expressed MEP pathway genes. The percentage survival was calculated based on the percentage of streaks that grew on selective plates.

Supplemental Figures

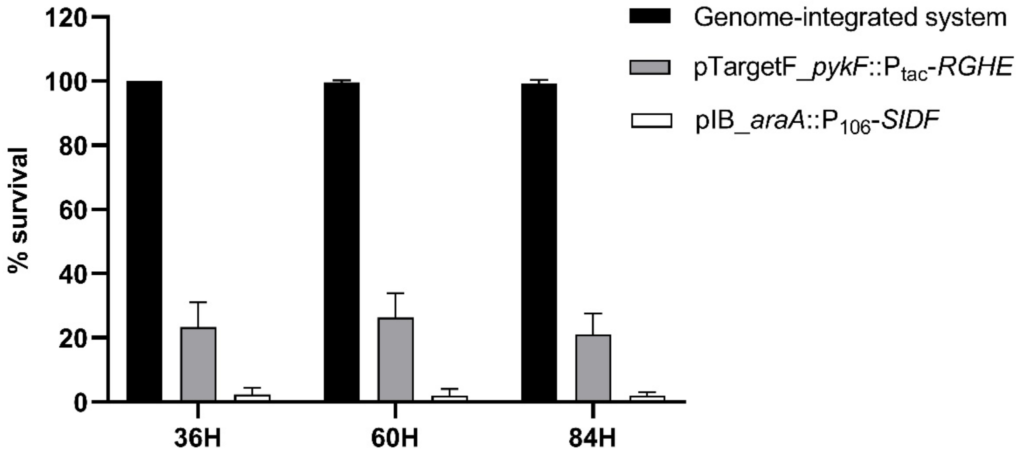

Fig.S1 Comparison of percentage survival of strains on the respective antibiotic selective media; the genome-integrated system: Nat^R; pIB_*araA::*P$_{106}$-*SIDF*: Cm^R; pTargetF_*pykF::P$_{tac}$-RGHE*: Sp^R. A greater loss of pIB_*araA::*P$_{106}$-*SIDF* was seen compared to pTargetF_*pykF::P$_{tac}$-RGHE* in the plasmid-based system. Error bars represent the average ± S.D. of three independent biological replicates.

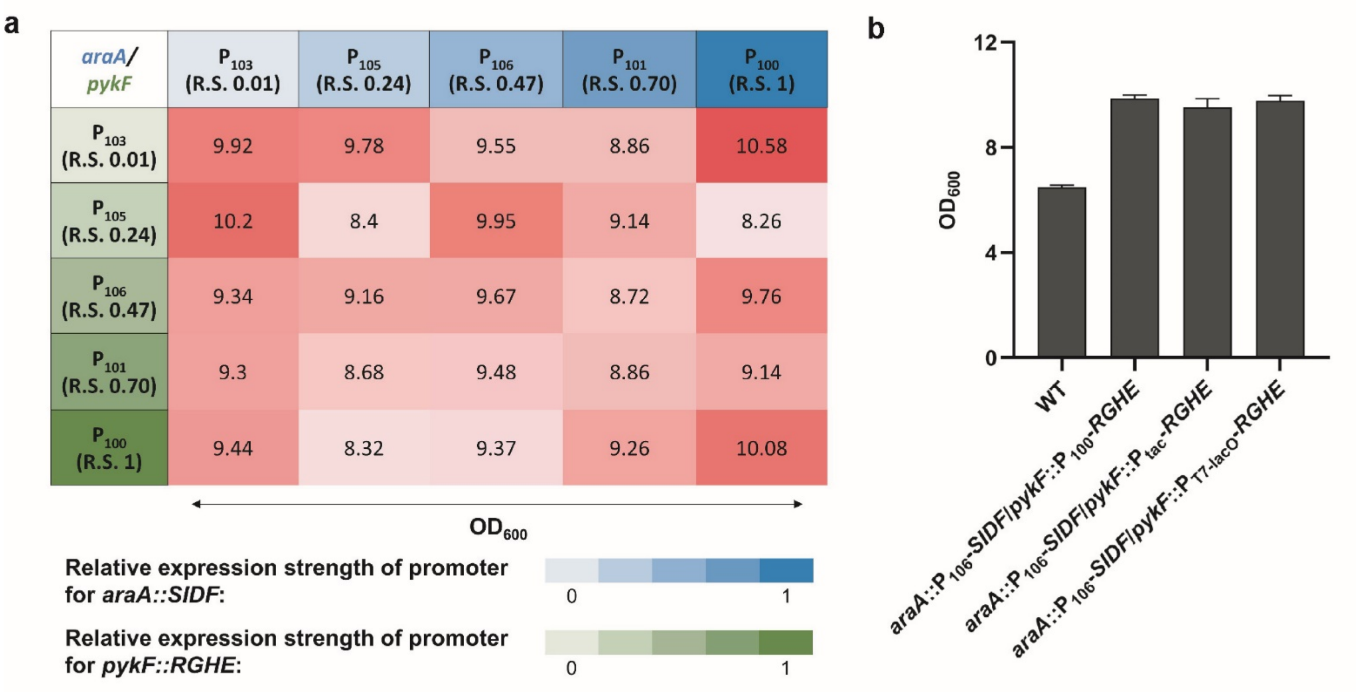

Fig.S2 Optical densities (OD_{600}) of (**a**) a combinatorial strain library expressing the heterologous MEP pathway genes from different IPTG-inducible Anderson promoters and (**b**) additional strains with IPTG-inducible P_{tac} and T7-lacO promoters. WT: wild type. Error bars represents the average ± S.D. of three independent biological replicates.

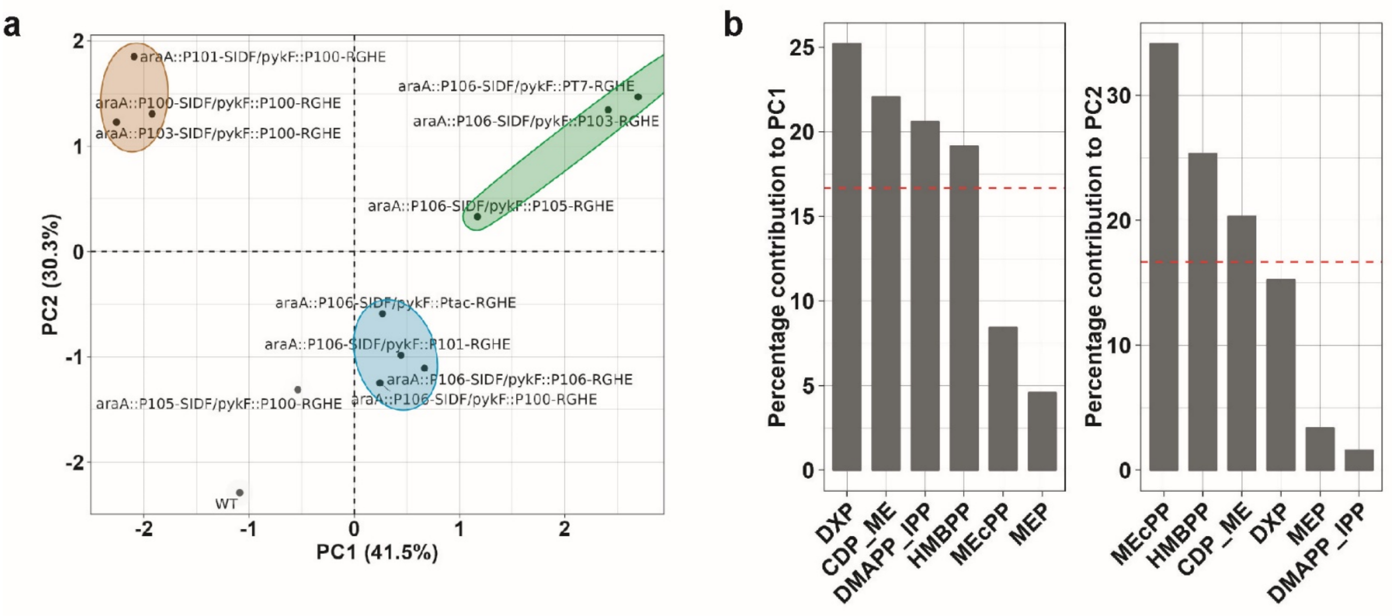

Fig.S3 Principal component analysis (PCA) of intracellular DXP, MEP, CDP-ME, MEcPP, HMBPP, and IPP/DMAPP levels in the engineered *E. coli* strains. **a.** Biplot of principal components (PC) 1 and 2 separating “top producers” (cyan ellipse), “medium producers” (green ellipse), and “low producers” (brown ellipse). WT: Wild type. **b.** Percentage contribution of each quantified metabolite to PC1 and PC2. The dashed line indicates the expected average contribution of six variables, which is 16.6%, the cutoff of importance.

Supplemental Tables

Table S1 List of strains used in this study.

Strain	Description	Source
DH5α	F-, 80dlacZM15, (*lacZYA-argF*)U169, *deoR*, *recA*1 *endA*1, *hsdR*17(rK- mK+), *phoA*, *supE*44, -, *thi*-1, gyrA96, *rel*A1	
MG1655(DE3)	F-, lamda-, *ilvG*-, *rfb*-50, *rph*-1	
*Ds*Red.GFPuv:2 plasmid system	MG1655(DE3) carrying pBluescript_P_{T7}-*Ds*Red and pCDF2_P_{tac}-GFPuv	This study
*Ds*Red.GFPuv:1 plasmid system, 200 copy	MG1655(DE3) carrying pBS_P_{T7}-*Ds*Red.GFPuv	This study
*Ds*Red.GFPuv:1 plasmid system, 15-20 copy	MG1655(DE3) carrying pTargetF_P_{T7}-*Ds*Red.GFPuv	This study
*Ds*Red.GFPuv: genome integrated	MG1655(DE3) with *DsRed* and *GFPuv* under a constitutive T7 promoter, integrated in the genome at the *araA* locus.	This study
WT	MG1655(DE3) carrying the pCDF2_P_{tac}-*ispA(S80F).tObGES*	This study
araA::P_{103}-*SIDF*/*pykF*::P_{103}-*RGHE*	MG1655(DE3) with the genome-integrated *araA*::*SIDF* under the K1585103 promoter and *pykF::RGHE* under the K1585103 promoter. The strain also carries the pCDF2_P_{tac}-*ispA(S80F).tObGES*.	This study
araA::P_{103}-*SIDF*/*pykF*::P_{105}-*RGHE*	MG1655(DE3) with the genome-integrated *araA*::*SIDF* under the K1585103 promoter and *pykF::RGHE* under the K1585105 promoter. The strain also carries the pCDF2_P_{tac}-*ispA(S80F).tObGES*.	This study
araA::P_{103}-*SIDF*/*pykF*::P_{106}-*RGHE*	MG1655(DE3) with the genome-integrated *araA*::*SIDF* under the K1585103 promoter and *pykF::RGHE* under the K1585106 promoter. The strain also carries the pCDF2_P_{tac}-*ispA(S80F).tObGES*.	This study
araA::P_{103}-*SIDF*/*pykF*::P_{101}-*RGHE*	MG1655(DE3) with the genome-integrated *araA*::*SIDF* under the K1585103 promoter and *pykF::RGHE* under the K1585101 promoter. The strain also carries the pCDF2_P_{tac}-*ispA(S80F).tObGES*.	This study
araA::P_{103}-*SIDF*/*pykF*::P_{100}-*RGHE*	MG1655(DE3) with the genome-integrated *araA*::*SIDF* under the K1585103 promoter and *pykF::RGHE* under the K1585100 promoter. The strain also carries the pCDF2_P_{tac}-*ispA(S80F).tObGES*.	This study
araA::P_{105}-*SIDF*/*pykF*::P_{103}-*RGHE*	MG1655(DE3) with the genome-integrated *araA*::*SIDF* under the K1585105 promoter and *pykF::RGHE* under the K1585103 promoter. The strain also carries the pCDF2_P_{tac}-*ispA(S80F).tObGES*.	This study
araA::P_{105}-*SIDF*/*pykF*::P_{105}-RGHE	MG1655(DE3) with the genome-integrated *araA*::*SIDF* under the K1585105 promoter and *pykF::RGHE* under the K1585105 promoter. The strain also carries the pCDF2_P_{tac}-*ispA(S80F).tObGES*.	This study
araA::P_{105}-SIDF/*pykF*::P_{106}-*RGHE*	MG1655(DE3) with the genome-integrated *araA*::*SIDF* under the K1585105 promoter and *pykF::RGHE* under the K1585106 promoter. The strain also carries the pCDF2_P_{tac}-*ispA(S80F).tObGES*.	This study
araA::P_{105}-*SIDF*/*pykF*::P_{101}-*RGHE*	MG1655(DE3) with the genome-integrated *araA*::*SIDF* under the K1585105 promoter and *pykF::RGHE* under the K1585101 promoter. The strain also carries the pCDF2_P_{tac}-*ispA(S80F).tObGES*.	This study

araA::P_{105}-*SIDF*/*pykF*::P_{100}-*RGHE*	MG1655(DE3) with the genome-integrated *araA*::*SIDF* under the K1585105 promoter and *pykF::RGHE* under the K1585100 promoter. The strain also carries the pCDF2_P_{tac}-*ispA(S80F).tObGES*.	This study
araA::P_{106}-*SIDF*/*pykF*::P_{103}-*RGHE*	MG1655(DE3) with the genome-integrated *araA*::*SIDF* under the K1585106 promoter and *pykF::RGHE* under the K1585103 promoter. The strain also carries the pCDF2_P_{tac}-*ispA(S80F).tObGES*.	This study
araA::P_{106}-*SIDF*/*pykF*::P_{105}-*RGHE*	MG1655(DE3) with the genome-integrated *araA*::*SIDF* under the K1585106 promoter and *pykF::RGHE* under the K1585105 promoter. The strain also carries the pCDF2_P_{tac}-*ispA(S80F).tObGES*.	This study
araA::P_{106}-*SIDF*/*pykF*::P_{106}-*RGHE*	MG1655(DE3) with the genome-integrated *araA*::*SIDF* under the K1585106 promoter and *pykF::RGHE* under the K1585106 promoter. The strain also carries the pCDF2_P_{tac}-*ispA(S80F).tObGES*.	This study
araA::P_{106}-*SIDF*/*pykF*::P_{101}-*RGHE*	MG1655(DE3) with the genome-integrated *araA*::*SIDF* under the K1585106 promoter and *pykF::RGHE* under the K1585101 promoter. The strain also carries the pCDF2_P_{tac}-*ispA(S80F).tObGES*.	This study
araA::P_{106}-*SIDF*/*pykF*::P_{100}-*RGHE*	MG1655(DE3) with the genome-integrated *araA*::*SIDF* under the K1585106 promoter and *pykF::RGHE* under the K1585100 promoter. The strain also carries the pCDF2_P_{tac}-*ispA(S80F).tObGES*.	This study
araA::P_{106}-*SIDF*/*pykF*::P_{tac}-*RGHE*	MG1655(DE3) with the genome-integrated *araA*::*SIDF* under the K1585106 promoter and *pykF::RGHE* under the P_{tac} promoter. The strain also carries the pCDF2_P_{tac}-*ispA(S80F).tObGES*.	This study
araA::P_{106}-*SIDF*/*pykF*::$P_{T7\text{-}lacO}$-*RGHE*	MG1655(DE3) with the genome-integrated *araA*::*SIDF* under the K1585106 promoter and *pykF::RGHE* under the IPTG inducible T7-lacO promoter. The strain also carries the pCDF2_P_{tac}-*ispA(S80F).tObGES*.	This study
araA::P_{101}-*SIDF*/*pykF*::P_{103}-*RGHE*	MG1655(DE3) with the genome-integrated *araA*::*SIDF* under the K1585101 promoter and *pykF::RGHE* under the K1585103 promoter. The strain also carries the pCDF2_P_{tac}-*ispA(S80F).tObGES*.	This study
araA::P_{101}-*SIDF*/*pykF*::P_{105}-*RGHE*	MG1655(DE3) with the genome-integrated *araA*::*SIDF* under the K1585101 promoter and *pykF::RGHE* under the K1585105 promoter. The strain also carries the pCDF2_P_{tac}-*ispA(S80F).tObGES*.	This study
araA::P_{101}-*SIDF*/*pykF*::P_{106}-*RGHE*	MG1655(DE3) with the genome-integrated *araA*::*SIDF* under the K1585101 promoter and *pykF::RGHE* under the K1585106 promoter. The strain also carries the pCDF2_P_{tac}-*ispA(S80F).tObGES*.	This study
araA::P_{101}-*SIDF*/*pykF*::P_{101}-*RGHE*	MG1655(DE3) with the genome-integrated *araA*::*SIDF* under the K1585101 promoter and *pykF::RGHE* under the K1585101 promoter. The strain also carries the pCDF2_P_{tac}-*ispA(S80F).tObGES*.	This study
araA::P_{101}-*SIDF*/*pykF*::P_{100}-*RGHE*	MG1655(DE3) with the genome-integrated *araA*::*SIDF* under the K1585101 promoter and *pykF::RGHE* under the K1585100 promoter. The strain also carries the pCDF2_P_{tac}-*ispA(S80F).tObGES*.	This study
araA::P_{100}-*SIDF*/*pykF*::P_{103}-*RGHE*	MG1655(DE3) with the genome-integrated *araA*::*SIDF* under the K1585100 promoter and *pykF::RGHE* under the K1585103 promoter. The strain also carries the pCDF2_P_{tac}-*ispA(S80F).tObGES*.	This study

araA::P_{100}-*SIDF*/*pykF*::P_{105}-*RGHE*	MG1655(DE3) with the genome-integrated *araA*::*SIDF* under the K1585100 promoter and *pykF::RGHE* under the K1585105 promoter. The strain also carries the pCDF2_P_{tac}-*ispA(S80F).tObGES*.	This study
araA::P_{100}-*SIDF*/*pykF*::P_{106}-*RGHE*	MG1655(DE3) with the genome-integrated *araA*::*SIDF* under the K1585100 promoter and *pykF::RGHE* under the K1585106 promoter. The strain also carries the pCDF2_P_{tac}-*ispA(S80F).tObGES*.	This study
araA::P_{100}-*SIDF*/*pykF*::P_{101}-*RGHE*	MG1655(DE3) with the genome-integrated *araA*::*SIDF* under the K1585100 promoter and *pykF::RGHE* under the K1585101 promoter. The strain also carries the pCDF2_P_{tac}-*ispA(S80F).tObGES*.	This study
araA::P_{100}-*SIDF*/*pykF*::P_{100}-*RGHE*	MG1655(DE3) with the genome-integrated *araA*::*SIDF* under the K1585100 promoter and *pykF::RGHE* under the K1585100 promoter. The strain also carries the pCDF2_P_{tac}-*ispA(S80F).tObGES*.	This study
araA::P_{106}-*SIDF*/*pykF*::P_{tac}-*RGHE-v2*	MG1655(DE3) with the genome-integrated *araA*::*SIDF* under the K1585106 promoter and *pykF::RGHE* under the P_{tac} promoter. The strain also carries the pCDFDuet1-kan^R_$P_{T7\text{-}lacO}$-*ispA(S80F).tObGES*	This study
Geraniol:3 plasmids	MG1655(DE3) transformed with plasmids pIB_*araA::*P_{106}-*SIDF,* pTargetF_*pykF::*P_{tac}-*RGHE,* and pCDFDuet1-kan^R_$P_{T7\text{-}lacO}$-*ispA(S80F).tObGES*	This study

Table S2 List of plasmids.

Plasmid	Description	Source
pBluescript KS II+	ColE1, *bla*, P_{T7}	Agilent
pCDFDuet1	CloDF13, *aadA*, *lacI^q*, $P_{T7\text{-}lacO}$	EMD Millipore
pCDFDuet1-kan^R	CloDF13, *kan^R*, *lacI^q*, $P_{T7\text{-}lacO}$	This study
pCDF2	CloDF13, *aadA*, *lacI^q*, P_{tac}	EMD Millipore
pKD46_*Cas9.RecA.Cure*	*SpCas9, PaRecA, repA101ts, bla, araC*, *araB*p-λ γ -λ β -λex	Gift from Dr. Quanjiang Ji
pYTK001	Rep_ori, *cml*, *sfGFP* dropout	(Lee et al. 2015)
pTargetF	pMB1, *aadA*, P_{J23119}	(Jiang et al. 2015)
pTargetF_*araA*	pTargetF containing a sgRNA to target the *araA* locus	This study
pTargetF_*pykF*	pTargetF containing a sgRNA to target the *pykF* locus	This study
pBluescript KS II+_P_{T7}-*DsRed*	pBluescript KS II+ expressing *DsRed* from a constitutive T7 promoter.	This study
pCDF2_P_{tac}-*GFPuv*	Plasmid pCDF2 expressing *GFPuv* from a P_{tac} promoter.	This study
pBluescript KS II+_P_{T7}-*DsRed.GFPuv*	pBluescript KS II+ expressing *DsRed* and *GFPuv* as an operon from a constitutive T7 promoter	This study
pTargetF_*araA::*P_{T7}-*DsRed.GFPuv*	pTargetF_*araA* modified to express *DsRed* (GenBank accession #: LT726797.1) and *GFPuv* (GenBank accession #: AJ310442.1) as an operon from a constitutive T7 promoter and the homology regions of the *araA* locus	This study
pCDF2_P_{tac}-*ispA(S80F).tObGES*	Plasmid pCDF2 expressing *ispA(S80F)* and *tObGES* from a double P_{tac} promoter	This study
pTargetF_*araA GG*	Plasmid pTargetF_*araA* modified to include two BsaI sites	This study
pTargetF_*pykF GG*	Plasmid pTargetF_*pykF* modified to include two BsaI sites	This study
pYTK001_*sfGFP dropout*	Plasmid pYTK001 expressing a *sfGFP* transcription unit for assembling pTargetF_*araA/pykF int GG1*	This study
pYTK001_*Kan^R dropout*	Plasmid pYTK001 contain a kanamycin resistant gene (*kan^r*) for assembling pTargetF_*araA/pykF int GG2*	This study
pYTK001_*sfGFP dropout2*	Plasmid pYTK001 modified to contain *sfGFP* (GenBank accession #: MK995039.1) with different overhangs than pYTK001_*sfGFP dropout* for assembling pTargetF_*araA/pykF int GG3*	This study
pYTK001_*LR-araA*	pYTK001 containing the 5' homology region of the *araA* locus, flanked by two BsaI sites	This study
pYTK001_*RR-araA*	pYTK001 containing the 3' homology region of the *araA* locus, flanked by two BsaI sites	This study
pYTK001_*dxs*	pYTK001 containing the dxs (EcoCyc accession #: G6237) flanked by two BsaI sites	This study
pYTK001_*idi*	pYTK001 containing *idi* (EcoCyc accession #: G7508) flanked by two BsaI sites	This study
pYTK001_*ispDF-term*	pYTK001 containing *ispDF* (EcoCyc accession #: G7423, EG11816) linked to L3S2P21 terminator sequence, flanked by two BsaI sites	This study
pYTK001_*NAT*	pYTK001 containing a nourseothricin resistant gene (*NAT^R*) (GenBank Accession #: MG897154.1) transcription unit flanked by two BsaI sites.	This study
pYTK001_*LR-pykF*	pYTK001 containing the 5' homology region of the *pykF* locus, flanked by two BsaI sites	This study
pYTK001_*RR-pykF*	pYTK001 containing the 3' homology region of the *pykF* locus, flanked by two BsaI sites	This study
pYTK001_*dxr*	pYTK001 containing the *dxr* (EcoCyc accession #: EG12715) flanked by two BsaI sites	This study

pYTK001_*ispG*	pYTK001 containing the *ispG* (EcoCyc accession #: EG10370) flanked by two BsaI sites	This study
pYTK001_*ispH*	pYTK001 containing the *ispH* (EcoCyc accession #: EG11081) flanked by two BsaI sites	This study
pYTK001_*ispE-term*	pYTK001 containing the *ispE* linked to T_{PheA} terminator, flanked by two BsaI sites	This study
pYTK001_P_{T7}-*lacI*	pYTK001 containing *lacI* (GenBank accession #: U73857.1) under a constitutive T7 promoter, flanked by two BsaI sites	This study
pYTK001_P_{tac}	pYTK001 containing an IPTG-inducible P_{tac} promoter, flanked by two BsaI sites	This study
pTargetF_*araA int GG1*	pTargetF_*araA GG* modified to contain the 5' homologous region for the *araA* locus, *sfGFP*, and the 3' homologous region for the *araA* locus. BsaI sites are at the 5' end of the 5' homology region and the 3' end of the 3' homology region.	This study
pTargetF_*araA int GG2*	pTargetF_*araA int GG1* with the *sfGFP* replaced by a kan^R, *ispDF* with its RBS and a terminator, and the nourseothricin resistant gene (NAT^R). BsaI sites are at the 5' end of the kan^R and 3' end of the NAT^R (GenBank Accession #: MG897154.1).	This study
pTargetF_*araA int GG3*	pTargetF_*araA int GG2* with the kan^R replaced by *sfGFP*, *dxs* with its RBS, and *idi* with its RBS. BsaI sites are at the 5' end of the *sfGFP* and 3' end of *idi*.	This study
pTargetF_*pykF int GG1*	pTargetF_*pykF GG* modified to encode the 5' homologous region for the *pykF* locus, *sfGFP*, and the 3' homology region for the *pykF* locus. BsaI sites are at the 5' end of the 5' homology region and the 3' end of the 3' homologous region.	This study
pTargetF_*pykF int GG2*	pTargetF_*pykF int GG1* with the *sfGFP* replaced by the kan^R, *ispH* with its RBS, and *ispE* with its RBS and terminator. BsaI sites are at the 5' end of the kan^R and 3' end of the terminator.	This study
pTargetF_*pykF int GG3*	pTargetF_*pykF int GG2* with the kan^R replaced by *sfGFP*, *dxr* with its RBS, and *ispG* with its RBS. BsaI sites are at the 5' end of the *sfGFP* and 3' end of *ispG*.	This study
pTargetF_ *araA::P_{103}-SIDF*	pTargetF_*araA int GG3* with the *sfGFP* replaced by *lacI* under a constitutive T7 promoter, and the K1585103 promoter.	This study
pTargetF_ *araA::P_{105}-SIDF*	pTargetF_*araA int GG3* with the *sfGFP* replaced by *lacI* under constitutive T7 promoter, and the K1585105 promoter.	This study
pTargetF_ *araA::P_{106}-SIDF*	pTargetF_*araA int GG3* with the *sfGFP* replaced by *lacI* under constitutive T7 promoter, and the K1585106 promoter.	This study
pTargetF_ *araA::P_{101}-SIDF*	pTargetF_*araA int GG3* with the *sfGFP* replaced by *lacI* under constitutive T7 promoter, and the K1585101 promoter.	This study
pTargetF_ *araA::P_{100}-SIDF*	pTargetF_*araA int GG3* with the *sfGFP* replaced by *lacI* under constitutive T7 promoter, and the K1585100 promoter.	This study
pTargetF_*pykF::P_{103}-RGHE*	pTargetF_*pykF int GG3* with the *sfGFP* replaced by *lacI* under constitutive T7 promoter, and the K1585103 promoter.	This study
pTargetF_ *pykF::P_{105}-RGHE*	pTargetF_*pykF int GG3* with the *sfGFP* replaced by *lacI* under constitutive T7 promoter, and the K1585105 promoter.	This study
pTargetF_ *pykF::P_{106}-RGHE*	pTargetF_*pykF int GG3* with the *sfGFP* replaced by *lacI* under constitutive T7 promoter, and the K1585106 promoter.	This study
pTargetF_ *pykF::P_{101}-RGHE*	pTargetF_*pykF int GG3* with the *sfGFP* replaced by *lacI* under constitutive T7 promoter, and the K1585101 promoter.	This study
pTargetF_ *pykF::P_{100}-RGHE*	pTargetF_*pykF int GG3* with the *sfGFP* replaced by *lacI* under constitutive T7 promoter, and the K1585100 promoter.	This study
pTargetF_ *pykF::P_{tac}-RGHE*	pTargetF_*pykF int GG3* with the *sfGFP* replaced by *lacI* under constitutive T7 promoter, and the P_{tac} promoter.	This study

pTargetF_ *pykF::$P_{T7-lacO}$-RGHE*	pTargetF_*pykF int GG3* with the *sfGFP* replaced by *lacI* under constitutive T7 promoter, and the IPTG-inducible T7-lacO promoter.	This study
pIB_ *araA::P_{106}-SIDF*	p15A, *cml*, *araA*::SIDF under the K1585106 promoter.	This study
pCDFDuet1-kan^R_$P_{T7-lacO}$-*ispA(S80F).tObGES*	Plasmid pCDFDuet1 modified to contain *kan^R*, and *ispA(S80F)* (GenBank accession #: OR123875) and codon-optimized *tObGES* (GenBank accession #: OR123874)under a T7-lacO promoter	This study

Table S3 List of primers for cloning.

Ribosome binding sequences (RBS) for all genes except *ispG*, *ispA(S80F)*, and t*Ob*GES are highlighted in the primer sequences.

RBS sequence for *ispG*: gccagccaataaggagatttcact

RBS sequence for *ispA(S80F)*: taagtatacaaaaattttaaagataaggaggtaaagt

RBS sequence for t*Ob*GES: cacaagcaataaggagcgattccat

Primer	Sequence 5' → 3' (RBS sequences highlighted)	Notes
*Ds*Red_RBS FW	tcggttaggagaagcagcccgcgctgagaaataaggaggtttttta tggacaacaccgaggac	For amplifying *DsRed* to clone pBluescript KS II+_P$_{T7}$-*DsRed*
*Ds*Red RW	tcactcgagctgggagcc	For amplifying *DsRed* to clone pBluescript KS II+_P$_{T7}$-*DsRed* cloning
pBS_*Ds*Red FW	ccggctcccagctcgagtgacgaattggagctccacc	For amplifying the plasmid backbone to clone pBluescript KS II+_ P$_{T7}$-*DsRed* cloning
pBS_*Ds*Red RW	gggctgcttctcctaaccgaccctatagtgagtcgtattacg	For amplifying the plasmid backbone to clone pBluescript KS II+_ P$_{T7}$-*DsRed* cloning
GFPuv_RBS FW	tggcagttgaactggatctgagatttaaggaggtattttatgagtaaa ggagaagaac	For amplifying *GFPuv* to clone pCDF2_P$_{tac}$-*GFPuv*
GFPuv RW	ttatttgtagagctcatcc	For amplifying *GFPuv* to clone pCDF2_P$_{tac}$-*GFPuv*
pCDF2_GFPuv FW	tggatgagctctacaaataattatttgccgactaccttg	For amplifying the pCDF2 backbone to clone pCDF2_P$_{tac}$-*GFPuv*
pCDF2_GFPuv RW	cagatccagttcaactgccagatcctgtttcctgtgtg	For amplifying the pCDF2 backbone to clone pCDF2_P$_{tac}$-*GFPuv*
pBS_GFPuv FW	tggatgagctctacaaataacgaattggagctccacc	For cloning pBluescript KS II+_P$_{T7}$-*DsRed.GFPuv*
dsRed_GFPuv RW	cagatccagttcaactgccatcactcgagctgggagcc	For cloning pBluescript KS II+_P$_{T7}$-*DsRed.GFPuv*
LR-araA FW	agtgcacgcagacatcc	For cloning pTargetF_*araA*::P$_{T7}$-*DsRed.GFPuv*
LR-araA_RR RW	cgatactgtcccacggcagcttacggtttgttgagcatggt	For cloning pTargetF_*araA*::P$_{T7}$-*DsRed.GFPuv*
RR-araA FW	gctgccgtgggacagtatcgatat	For cloning pTargetF_*araA*::P$_{T7}$-*DsRed.GFPuv*
RR-araA RW	aggtggtggtgaacgcgtg	For cloning pTargetF_*araA*::P$_{T7}$-*DsRed.GFPuv*
pTargetF_RR FW	ccacgcgttcaccaccacctatctattaccctgttatccc	For cloning pTargetF_*araA*::P$_{T7}$-*DsRed.GFPuv*
pTargetF_LR RW	atgggatgtctgcgtgcactctaagcttctgcaggt	For cloning pTargetF_*araA*::P$_{T7}$-*DsRed.GFPuv*
RR_pTar_GFPuv FW	tggatgagctctacaaataagctgccgtgggacagtatcgatat	For cloning pTargetF_*araA*::P$_{T7}$-*DsRed.GFPuv*
LR_pTar_*Ds*Red RW	cctatagtgagtcgtattacttacggtttgttgagcatggtcagg	For cloning pTargetF_*araA*::P$_{T7}$-*DsRed.GFPuv*
pTargetF-sgRNA FW	P-attccacacccagttcaacggttttagagctagaaatagc	For cloning pTargetF_*araA*

pTar-pykF sgRNA FW(P)	P-gagcacctgaaagcgcacgggttttagagctagaaatagc	For cloning pTargetF_*pykF*
pTargetF GG FW (P)	P-tttgacggtctctccgaattaccctg	For cloning pTargetF_*araA GG* and pTargetF_*pykF GG*
pTargetF GG RW (P)	P-tttgatggtctctactcctaagcttctg	For cloning pTargetF_*araA GG* and pTargetF_*pykF GG*
pYTK001 FW GG	tttcgtctctgaccagaccaataaaaaacgccc	For amplifying the pYTK001 backbone to clone pYTK001_*sfGFP dropout*
pYTK001 RW GG	tttcgtctcgccgactacggttatccacagaatc	For amplifying the pYTK001 backbone to clone pYTK001_*sfGFP dropout*
sfGFP FW GG	tttcgtctcgtcggccctagagaccgacggaaagtgaaacgtgatttcat	For cloning pYTK001_*sfGFP dropout*
sfGFP RW GG	tttcgtctctggtctgtatgagaccgacgtataaacgcagaaaggcc	For cloning pYTK001_sfGFP dropout
KanR FW GG	tttcgtctcgtcggccctagagacccaccgacgtcggaattgc	For cloning pYTK001_*Kan^R dropout*
KanR RW GG	tttcgtctctggtcggattgagacctgactagtgcttggattctcacc	For cloning pYTK001_ *Kan^R dropout*
sfGFP RW GG2	tttcgtctctggtcgccatgagaccgacgtataaacgcagaaaggcc	For cloning pYTK001_sfGFP dropout2
araA-LR FW	tttcgtctcgtcggggtctcggagtagtgcacgcagacat	For cloning pYTK001_*LR-araA*
araA-LR RW	tttcgtctctggtcggtctctagggttacggtttgttgagcatg	For cloning pYTK001_*LR-araA*
RR-araA FW GG	tttcgtctcgtcggggtctcgtacagctgccgtgggacagta	For cloning pYTK001_*RR-araA*
RR-araA mut RW	tttcgtctcccgcctgacgcatcca	For domesticating *araA* type II restriction sites to clone pYTK001_*RR-araA*
RR-araA mut FW	tttcgtctcaggcggtatctaaacaggata	For domesticating *araA* type II restriction sites to clone pYTK001_*RR-araA*
RR-araA RW GG	tttcgtctctggtcggtctcttcggaggtggtggtgaacgc	For cloning pYTK001_*RR-araA*
dxs FW GG	tttcgtctcgtcggggtctcatggcgagcggacaaccgaatttagttaaggagcactttatgagttttgatattgccaaataccc	For cloning pYTK001_*dxs*
dxs RW GG	tttcgtctctggtcggtctctcgttttatgccagccaggcct	For cloning pYTK001_*dxs*
idi FW GG	tttcgtctcgtcggggtctcgaacgggaataatagtaaggaaaggtaatttaatgcaaacggaacacgt	For cloning pYTK001_*idi*
idi RW GG	tttcgtctctggtcggtctctggatttatttaagctgggtaaatgcag	For cloning pYTK001_*idi*
ispDF FW GG	tttcgtctcgtcggggtctcgatccacgagctagttaataatatataaggaggtatttgatggcaaccactcatttgga	For cloning pYTK001_*ispDF-term*
ispDF-term RW GG	tttcgtctctggtcggtctctcagcggaccaaaacgaaaaaaggccccccttcggggaggcctcttttctggaatttggtaccgagtcattttgttgccttaatgagtagc	For cloning pYTK001_*ispDF-term*
NAT FW GG	tttcgtctcgtcggggtctcggctgtaggtctagagatctgtttagcttg	For cloning pYTK001_*NAT*
NAT mut RW	tttcgtctcgagtacgatacgaccacga	For domesticating *NAT* type II restriction sites to clone pYTK001_*NAT*
NAT mut FW	tttcgtctcgtactccggctg	For domesticating *NAT* type II restriction sites to clone pYTK001_*NAT*
NAT RW GG	tttcgtctctggtcggtctcttgtaattaagggttctcgagagctc	For cloning pYTK001_*NAT*

pykF-LR FW	gattctgtggataaccgtagggtctcggagtgaacttctctcatggtgactatgc	For cloning pYTK001_*LR-pykF*
pykF-LR RW	cgttttttattggtctggtcggtctctagggttagatttcgataacgtcagaacgc	For cloning pYTK001_*LR-pykF*
pykF-RR FW	gattctgtggataaccgtagggtctcgtacagaaaacatccacatcatctcc	For cloning pYTK001_*RR-pykF*
pykF-RR RW	cgttttttattggtctggtcggtctcttcggatttaccgccctgagtag	For cloning pYTK001_*RR-pykF*
dxr FW GG2	tttcgtctcgtcggggtctcatatgagctacttggataacatacggaggaaaactatgaagcaactcaccattctgg	For cloning pYTK001_*dxr*
dxr mut RW	tttcgtctccaatggcgtttcacggaaag	For domesticating *dxr* type II restriction sites to clone pYTK001_*dxr*
dxr mut FW	tttcgtctcccattgcgcgatttggcaacaatgacg	For domesticating *dxr* type II restriction sites to clone pYTK001_*dxr*
dxr RW GG	tttcgtctctggtcggtctctcgtttcagcttgcaagacgcat	For cloning pYTK001_*dxr*
ispG FW GG2	tttcgtctcgtcggggtctcgaacggccagccaataaggagatttc	For cloning pYTK001_*ispG*
ispG RW GG2	tttcgtctctggtcggtctctggatttatttttcaacctgctgaacgt	For cloning pYTK001_*ispG*
ispH FW	gattctgtggataaccgtagggtctcgatcctaagaaatcgactaaggacaatttacatgcagatcctgttggcc	For cloning pYTK001_*ispH*
ispH RW	cgttttttattggtctggtcggtctctcagcttaatcgacttcacgaatatcgac	For cloning pYTK001_*ispH*
ispE FW	tttcgtctcgtcggggtctcggctgcaataaaacgataagaagaggcaatttatgcggacacagtggccctctccg	For cloning pYTK001_*ispE-term*
ispE mut RW	tttcgtctcagaccgccgcccatcggcaaa	For domesticating *ispE* type II restriction sites to clone pYTK001_*ispE-term*
ispE mut FW	tttcgtctccggtctaggcggtggttcat	For domesticating *ispE* type II restriction sites to clone pYTK001_*ispE-term*
ispE-term RW	tttcgtctctggtcggtctcttgtattttgttatcaataaaaaaggcccccgatttgggaggccttattgttcgtcttaaagcatggctctgtgcaatgggg	For cloning pYTK001_*ispE-term*
LacI-T7 FW Gib	gattctgtggataaccgtagggtctcgccctgtaatacgactcactataggggaggtaaagaacccggaggaggtattttcgtgaaaccagtaacgttatacgatg	For cloning pYTK001_P_{T7}-*lacI*
LacI RW Gib	cgttttttattggtctggtcggtctctcatagaaccgttatgatgtcggcg	For cloning pYTK001_P_{T7}-*lacI*
tac FW GG 3	tttcgtctcgtcggggtctcatatggagctgttgacaattaatcatcggctcg	For cloning pYTK001_P_{tac}
tac RW GG	tttcgtctctggtcggtctcagccagatcctgtttcctgtgtg	For cloning pYTK001_P_{tac}
T7 FW GG	tttcgtctcgtcggggtctcatatgttaatacgactcactataggggaattgtgagcggataacaattccctggctgagaccgaccagagacgaa	Oligo for pYTK001_$P_{T7\text{-}lacO}$. The *lacO* region is underlined.
T7 RW GG	tttcgtctctggtcggtctcagccagggaattgttatccgctcacaattcccctatagtgagtcgtattaacatatgagaccccgacgagacgaaa	Oligo for pYTK001_$P_{T7\text{-}lacO}$
K1585103 double-stranded oligo	tttcgtctcgtcggggtctcatatgctgatagctagctcagtcctagggattatgctagctactagagtgtgtggaattgtgagcggataacaatttcacacatggctgagaccgaccagagacgaaa	Oligo for K1585103 promoter. The *lacO* region is underlined.
K1585105 double-stranded oligo	tttcgtctcgtcggggtctcatatgtttacggctagctcagtcctaggtactatgctagctactagagtgtgtggaattgtgagcggataacaatttcacacatggctgagaccgaccagagacgaaa	Oligo for K1585105 promoter. The *lacO* region is underlined.

K1585106 double-stranded oligo	tttcgtctcgtcggggtctcatatgtttacggctagctcagtcctaggtatagtgctagctactagagtgtgtggaattgtgagcggataacaatttcacacatggctgagaccgaccagagacgaaa	Oligo for K1585106 promoter. The *lacO* region is underlined.
K1585101 double-stranded oligo	tttcgtctcgtcggggtctcatatgtttacagctagctcagtcctaggtattatgctagctactagagtgtgtggaattgtgagcggataacaatttcacacatggctgagaccgaccagagacgaaa	Oligo for K1585101 promoter. The *lacO* region is underlined.
K1585100 double-stranded oligo	tttcgtctcgtcggggtctcatatgttgacggctagctcagtcctaggtacagtgctagctactagagtgtgtggaattgtgagcggataacaatttcacacatggctgagaccgaccagagacgaaa	Oligo for K1585100 promoter. The *lacO* region is underlined.
p15A-Cm FW Gib	tattgagctggggatgaagccctcgctctgctaatcctgttac	For cloning pIB_*araA::*P_{106}*-SlDF*
p15A-Cm RW Gib	caggtcgactctagagaattgatcgggctcgccacttc	For cloning pIB_*araA::*P_{106}*-SlDF*
Km FW Gib	tcacactgcttccggtagtccaccgacgtcggaattg	For cloning pCDFDuet1-kan^R
Km RW Gib	ccgagtgagctagctatttgctagtgcttggattctcacc	For cloning pCDFDuet1-kan^R
pCDFDuet1 FW	caaatagctagctcactcgg	For cloning pCDFDuet1-kan^R
pCDFDuet1 RW	gactaccggaagcagtgt	For cloning pCDFDuet1-kan^R
ispA* FW Gib	caccatcatcaccacagccaggatcctaagtatacaaaaattttaaag	For cloning pCDFDuet1-kan^R_$P_{T7\text{-}lacO}$-*ispA(S80F).tObGES*
*Ob*GES RW Gib	ggtttctttaccagactcgaaagcttttattgtgtaaaaaacagggc	For cloning pCDFDuet1-kan^R_$P_{T7\text{-}lacO}$-*ispA(S80F).tObGES*

Table S4 List of primers for genotyping.

Primer	5' → 3' sequence	Purpose	Notes
gen CmR FW	aaccaggtcattatgcaggc	Forward primer to screen for the 5' end of *araA*::SIDF	Binds to the genome region upstream of *araA::SIDF*
gen CmR RW	caggcgttacataccggatg	Reverse primer to screen for the 3' end of *araA*::SIDF	Binds to the genome region downstream of *araA::SIDF*
dxs RW_short	ttatgccagccaggccttg	Reverse primer to screen for the 5' end of *araA*::SIDF	Binds to the *dxs* coding region
pBS-araA seq P9 FW	gccatatggatgatgttaacgt	Forward primer to screen for the 3' end of *araA*::SIDF	Binds to the *ispF* coding region
gen. pykF FW	gctggcatgaacgttatgc	Forward primer to screen for the 5' end of the *pykF*::RGHE	Binds to the genome region upstream of *pykF::RGHE*
gen. pykF RW2	gcagaacgttcagacaatgc	Reverse primer to screen for the 3' end of the *pykF*::RGHE	Binds to the genome region downstream of *pykF::RGHE*
dxr RW_screen2	tcgtccattacggcatagc	Reverse primer to screen for the 5' end of the *pykF*::RGHE	Binds to the *dxr* coding region
ispE FW_screen2	gatacagagtctgaagcccg	Forward primer to screen for the 3' end of the *pykF*::RGHE	Binds to the *ispE* coding region

Table S5 sgRNA protospacer sequences.

Target locus	5' →3' sequence
araA	attccacacccagttcaacg
pykF	gagcacctgaaagcgcacgg

Table S6 Homology regions for integration of MEP genes at *araA* and *pykF* genomic loci

Homology region	Sequence
5' homology region of *araA* locus	agtgcacgcagacatcccatcagctcagcaaaaaatggccagtgcggtagagaaaccctgcaaccgtgcagcgagcaggcacaacgctttgaacagctttatcgccgctatcagcaatgggcgatgagcgccgaacaacactatcttccaacttccgccccggcacaggctgcccaggccgttgcgactctataaggacacgataatgacgatttttgataattatgaagtgtggtttgtcattggcagccagcatctgtatggcccggaaaccctgcgtcaggtcacccaacatgccgagcacgtcgttaatgcgctgaatacggaagcgaaactgccctgcaaactggtgttgaaaccgctgggcaccacgccggatgaaatcaccgctatttgccgcgacgcgaattacgacgatcgttgcgctggtctggtggtgtggctgcacaccttctccccggccaaaatgtggatcaacggcctgaccatgctcaacaaaccgt
3' homology region of *araA* locus	gctgccgtgggacagtatcgatatggactttatgaacctgaaccagactgcacatggcggtcgcgagttcggcttcattggcgcgcgtatgcgtcagcaacatgccgtggttaccggtcactggcaggataaacaagcccatgagcgtatcggctcctggatgcgtcaggcggtctctaaacaggatacccgtcatctgaaagtctgccgatttggcgataacatgcgtgaagtggcggtcaccgatggcgataaagttgccgcacagatcaagttcggtttctccgtcaatacctgggcggttggcgatctggtgcaggtggtgaactccatcagcgacggcgatgttaacgcgctggtcgatgagtacgaaagctgctacaccatgacgcctgccacacaaatccacggcaaaaaacgacagaacgtgctggaagcggcgcgtattgagctggggatgaagcgtttcctggaacaaggtggcttccacgcgttcaccaccacct
5' homology region of *pykF* locus	gaacttctctcatggtgactatgcagaacacggtcagcgcattcagaatctgcgcaacgtgatgagcaaaactggtaaaaccgccgctatcctgcttgataccaaaggtccggaaatccgcaccatgaaactggaaggcggtaacgacgtttctctgaaagctggtcagacctttactttcaccactgataaatctgttatcggcaacagcgaaatggttgcggtaacgtatgaaggtttcactactgacctgtctgttggcaacaccgtactggttgacgatggtctgatcggtatggaagttaccgccattgaaggtaacaaagttatctgtaaagtgctgaacaacggtgacctgggcgaaaacaaaggtgtgaacctgcctggcgtttccattgctctgccagcactggctgaaaaagacaaacaggacctgatctttggttgcgaacaaggcgtagactttgttgctgcttcctttattcgtaagcgttctgacgttatcgaaatc
3' homology region of *pykF* locus	gaaaacatccacatcatctccaaaatcgaaaaccaggaaggcctcaacaacttcgacgaaatcctcgaagcctctgacggcatcatggttgcgcgtggcgacctgggtgtagaaatcccggtagaagaagttatcttcgcccagaagatgatgatcgaaaaatgtatccgtgcacgtaaagtcgttatcactgcgacccagatgctggattccatgatcaaaaacccacgcccgactcgcgcagaagccggtgacgttgcaaacgccatcctcgacggtactgacgcagtgatgctgtctggtgaatccgcaaaaggtaaatacccgctggaagcggtttctatcatggcgaccatctgcgaacgtaccgaccgcgtgatgaacagccgtctcgagttcaacaatgacaaccgtaaactgcgcattaccgaagcggtatgccgtggtgccgttgaaactgctgaaaaactggatgctccgctgatcgtggttgctactcagggcggtaaat

Table S7 list of primers for qRT-PCR.

Primer	5' →3' sequence
idnT F	gctgcttcttatcctgatgatcg
idnT R	gtatagagtgcaggacggc
dxs F	ctggcactggtcgactc
dxs R	gaacggctcacgctgtc
dxr F	gtcactcgcatggtagaacag
dxr R	ccacttaagacttcggtgcg
ispD F	accattcttgaacactcggtg
ispD R	ctacaacggtgatttgcgga
ispE F v2	ttcttgattacggcgacacc
ispE R v2	cgcgaacgatcaggttatcttc
ispF F v2	ttggcaacgtcgatgtcac
ispF R v2	gagatcttcggcaataaacacg
ispG F	cagacgtcgaagcaacgg
ispG R	cggcacgttaacctgctg
ispH F	gtccgtcacgaagtggtac
ispH R	cgttacgtaccgcctgag
idi F	ggcagacacccgcttac
idi R	gagttagtccacacgccag

Supplemental codes

R code for principal component analysis (PCA)

```
> library("FactoMineR")

> install.packages("devtools")

> library("devtools")

> install_github("kassambara/factoextra")

> library("factoextra")

> metabolites_data <- read.csv("D:\\Asus laptop complete backup\\D drive_New Volume\\Wang lab\\MEP project data\\MEP intermediates LCMS\\20221025 Intermediate quantification\\Integrated again and quantified\\metabolites.csv")          #reads the .csv file

> new_metabolites_dataset <- metabolites_data[,-1]          #reads the file without the first column

> rownames(new_metabolites_dataset) <- metabolites_data[,1]   #reads only the first column to assign "row names"

> View(new_metabolites_dataset)          #displays how the file is being read

> pca <- PCA(new_metabolites_dataset, graph = FALSE)          #correlation-based principal component analysis with scaling to unit variance

> fviz_pca_ind(pca, repel = TRUE)          #displays biplot

> fviz_eig(pca, addlabels = TRUE)          #displays scree plot

> var <- get_pca_var(pca)          #extracts the results for individual parameters of PCA

> a<-fviz_contrib(pca, choice = "var", axes = 1)          #assigns PC1 individual contributors graph to a variable

> b<-fviz_contrib(pca, choice = "var", axes = 2)          #assigns PC2 individual contributors graph to a variable

> install.packages("gridExtra")

> library(gridExtra)

> grid.arrange(a,b, ncol=2)          #displays PC1 and PC2's individual contributors
```

Appendix I

4-nt overhangs used for Golden Gate assembly of MEP pathway genes:

ccct, tggc, aacg, atcc, gctg, taca, ccga

These overhangs are from left to right in the cloned plasmids.

References:

Jiang Y, Chen B, Duan C, Sun B, Yang J, Yang S (2015) Multigene editing in the *Escherichia coli* genome via the CRISPR-Cas9 system. Appl Environ Microbiol 81(7):2506-2514 doi:10.1128/AEM.04023-14

Lee ME, DeLoache WC, Cervantes B, Dueber JE (2015) A highly characterized yeast toolkit for modular, multipart assembly. ACS Synth Biol 4(9):975-986 doi:10.1021/sb500366v
